# Critical Loss of the Balance between Th17 and T Regulatory Cell Populations in Pathogenic SIV Infection

**DOI:** 10.1371/journal.ppat.1000295

**Published:** 2009-02-13

**Authors:** David Favre, Sharon Lederer, Bittoo Kanwar, Zhong-Min Ma, Sean Proll, Zeljka Kasakow, Jeff Mold, Louise Swainson, Jason D. Barbour, Carole R. Baskin, Robert Palermo, Ivona Pandrea, Christopher J. Miller, Michael G. Katze, Joseph M. McCune

**Affiliations:** 1 Department of Medicine, Division of Experimental Medicine, University of California, San Francisco, California, United States of America; 2 Department of Microbiology, University of Washington, Seattle, Washington, United States of America; 3 Department of Pediatrics, Division of Gastroenterology, Hepatology, and Nutrition, University of California, San Francisco, California, United States of America; 4 Center for Comparative Medicine, California National Primate Research Center, University of California, Davis, California, United States of America; 5 Department of Medicine, HIV/AIDS Division, University of California, San Francisco, California, United States of America; 6 Washington National Primate Research Center, University of Washington, Seattle, Washington, United States of America; 7 Tulane National Primate Research Center, Covington, Louisiana, United States of America; University of Geneva, Switzerland

## Abstract

Chronic immune activation and progression to AIDS are observed after SIV infection in macaques but not in natural host primate species. To better understand this dichotomy, we compared acute pathogenic SIV infection in pigtailed macaques (PTs) to non-pathogenic infection in African green monkeys (AGMs). SIVagm-infected PTs, but not SIVagm-infected AGMs, rapidly developed systemic immune activation, marked and selective depletion of IL-17-secreting (Th17) cells, and loss of the balance between Th17 and T regulatory (Treg) cells in blood, lymphoid organs, and mucosal tissue. The loss of Th17 cells was found to be predictive of systemic and sustained T cell activation. Collectively, these data indicate that loss of the Th17 to Treg balance is related to SIV disease progression.

## Author Summary

Natural infection by the simian immunodeficiency virus (SIV) in over 40 different species of African non-human primates is not accompanied by progression to acquired immunodeficiency syndrome (AIDS). To understand this phenomenon, we have performed a detailed virologic, immunologic, and gene expression analysis of acute SIV infection of two disparate species: the African green monkey (AGM), in which SIV infection is nonpathogenic, and the Asian pigtailed macaque (PT), in which SIV infection results in AIDS. After experimental infection, animals of both species developed high viral loads. In the PTs, viremia was associated with CD4^+^ T cell depletion in the peripheral blood and multiple signs of persistent immune activation and inflammation. Such pathology was not observed in AGMs. Notably, the AGMs maintained high and balanced levels of two subset populations of CD4^+^ T cells, e.g., the immunosuppressive T regulatory (Treg) and the IL-17 producing (Th17) populations, whereas the PTs did not. Further analysis of the role of Th17 and Treg balance during pathogenic lentiviral infection may provide novel insights into our understanding of SIV and HIV pathogenesis and future thoughts about vaccine development.

## Introduction

Progressive disease caused by the human immunodeficiency virus, type 1 (HIV) is marked by systemic inflammation and immune dysregulation, most notably CD4^+^ T cell depletion [1],[2],[3],[4]. Insights into HIV-induced pathogenesis have been provided by studying SIV infection of non-human primates [5], wherein outcomes can be diametrically opposed depending on the species used. Thus, SIV infection of macaques leads to a disease syndrome indistinguishable from that induced by HIV in humans [6],[7],[8]. By contrast, SIV infection of the African green monkey (AGM) results in high viral loads in blood and lymphoid tissues, but limited inflammation and chronic immune activation, and no pathology [6],[9],[10],[11],[12].

Although it is not clear why some lentiviral infections trigger high levels of inflammation while others do not, it is evident that decisive host-pathogen interactions take place early after infection and can be determinants of disease progression. Acute HIV infection is associated with an early peak in viremia followed by partial resolution and the establishment of relatively stable “set points” in viral load and immune activation. Of these two parameters, the level of immune activation has been found to most accurately predict the rate of subsequent disease progression, independently of viral load [13],[14],[15]. The mechanisms by which this “immune activation set point” is established after acute infection have been difficult to elucidate. It is known, for example, that pathogenic SIV and HIV infections lead to rapid depletion of CD4^+^ T cells in the lamina propria of the gut, with impaired integrity of the mucosal epithelium, enhanced bacterial translocation, and perhaps systemic immune activation [16],[17]. However, acute CD4^+^ T cell depletion in the gut also occurs in the setting of “nonpathogenic” SIV infection [18],[19], raising the question as to when and how differential host responses to lentiviral infection are in fact established.

To more carefully define how lentiviral infections may cause immune activation and disease, we investigated the early events that occur in blood and hematolymphoid organs after pathogenic and nonpathogenic SIV infection. We hypothesized that a critical distinction between pathogenic and nonpathogenic infections may lie in a shift in the equilibrium between pro- and anti-inflammatory host immune responses during acute infection. Because helper CD4^+^ T cells orchestrate critical functions in the immune system through the production of distinct cytokine profiles [20], we investigated more specifically the relative frequency of subset populations of T helper cells, including two unique subsets under balanced and reciprocal patterns of differentiation: Th17 cells, producing the proinflammatory cytokine IL-17, and Tregs, whose function is instead immunosuppressive [21],[22]. Th17 cells, in particular, have been causally related both to chronic inflammatory diseases [23] and to host defenses against microbial agents [24]. Our data collectively indicate that pathogenic SIV infection results in the loss of balance between Th17 and Treg populations, whereas this balance is maintained in nonpathogenic infections.

## Results

### SIV infection of the PT, but not the AGM, results in T cell activation and generalized CD4+ T cell depletion

The primary isolate SIVagm.Sab92018 was used to infect (IV, 600 TCID_50_) four pigtailed macaques (PT; *Macaca nemestrina*) and four African green monkeys (AGM; *Chlorocebus sabaeus*). This primary isolate is derived from a wild-caught chronically SIV-infected *C. sabaeus* AGM from Senegal [25] and causes AIDS in 50% of infected pigtailed macaques within two years (I. Pandrea, manuscript in preparation), consistent with a prior report establishing SIV infection of pigtailed macaques with SIVagm isolates to be pathogenic [7]. Peripheral blood was obtained at time points before (days −14, 0) and after (days 3, 10, 17, 28, and 45 or 49) infection; lymph node, bone marrow, and colon were biopsied on days −14 and 10, and harvested at necropsy on days 45 or 49 (subsequently referred to as day “45+”). To define the relative importance of pro- versus anti-inflammatory host immune responses in pathogenic and nonpathogenic infections, samples were processed for comprehensive virologic, immunologic, histologic, and gene expression analyses.

**Figure 1:**
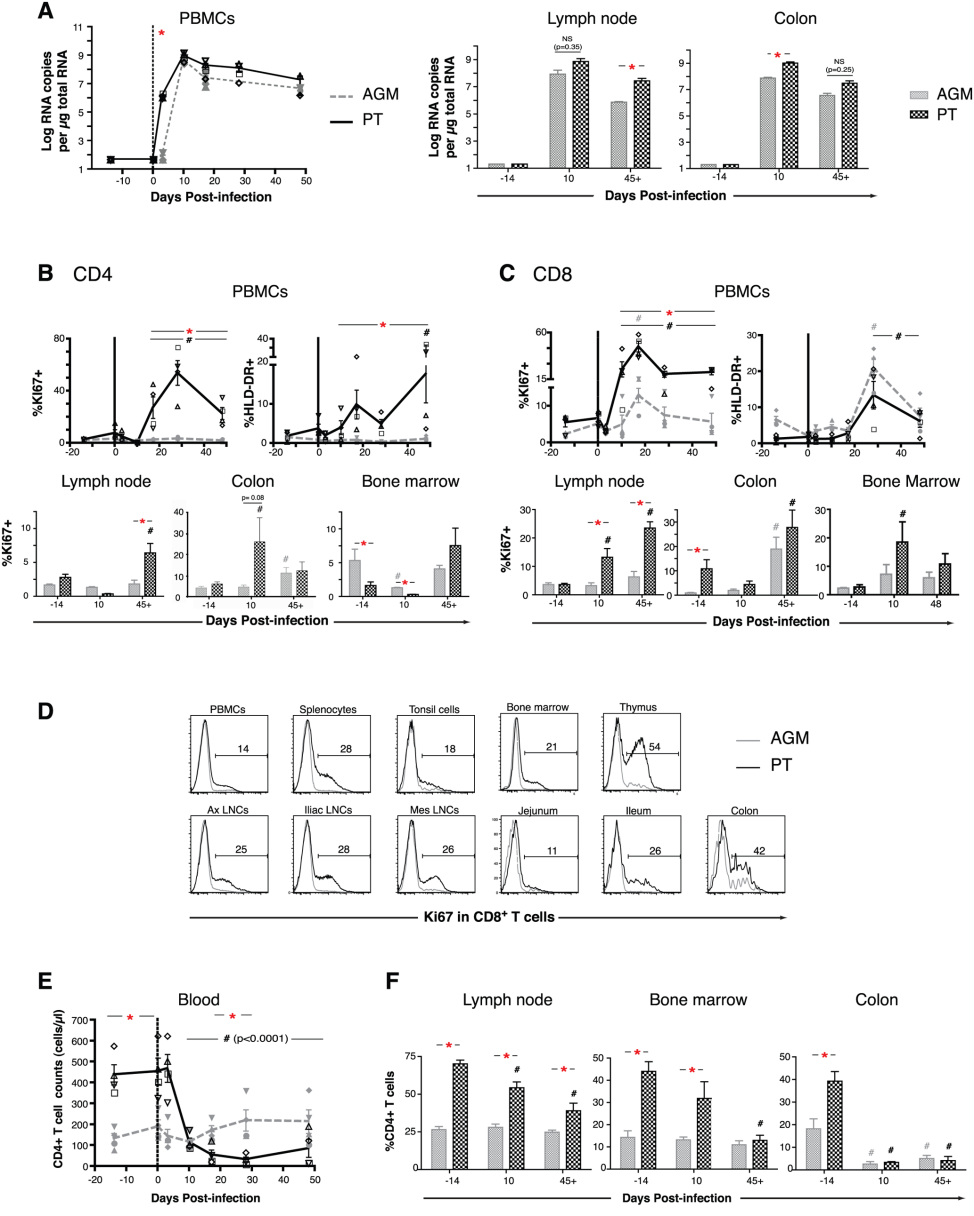
Virus load, T cell activation, and CD4^+^ and CD8^+^ T cell changes after SIV infection of PTs and AGMs. (A) Cellular RNA virus load in PBMCs, lymph node, and colon (per µg of total RNA), before and after inoculation of 4 PTs and 4 AGMs with the SIVagm isolate, SIVagm.sab92018 (600 TCID_50_). (B and C) Fraction of cells positive for Ki67 and HLA-DR amongst total CD4^+^ (B) and CD8^+^ (C) T cells from peripheral blood (PBMCs), lymph node, colon, and bone marrow. (D) Ki67^+^CD8^+^ T cells in multiple hematolymphoid organs at necropsy in a representative PT (A99059) and AGM (06006), with the frequency of Ki67^+^ cells indicated for the PT. (E) CD4^+^ T cell counts in the peripheral blood as a function of time after infection. (F) Frequency of CD4^+^ T cells in biopsies of lymph node, bone marrow, and colon, obtained at the indicated times before and after infection. Data are presented as means±SEM. Statistical analysis: *P* values were obtained on a per group basis (*) using the Mann-Whitney nonparametric test (when comparing PTs to AGMs at a given time point) or over time (#) by ANOVA (linear scale) (comparing each time point separately to baseline, e.g. day −14 and day 0, in PTs or AGMs). When the *p* value is not indicated on the graph, “*” or “#,” indicate that *p*<0.05 for each test, respectively.

In both species, and as expected [26], SIV viremia was detectable in PTs and AGMs by day 3 post-infection and reached high peak levels by day 10 (Figure S1A), after which it declined and remained stable at high viral “set-points” until the termination of the experiment (day 45+). The plasma viral load in the PT was consistently higher than that found in the AGM (p<0.02). Within cells from various anatomic compartments, however, viral loads in the PT were found to be higher only at scattered time points, e.g., at day 3 in peripheral blood mononuclear cells (PBMCs), at day 45+ in the lymph node, and at day 10 in the colon (Figure 1A).

Notwithstanding the similar kinetics of virus replication post-infection, PTs and AGMs showed different patterns of T cell activation. In the PT but not the AGM, CD4^+^ T cell activation (Ki67^+^ and HLA-DR^+^) was robust and sustained in PBMCs, lymph node, and bone marrow (Figures 1B and S1B), and persisted in the colon of both species until day 45+ (Figure 1B, lower). In CD8^+^ T cells from peripheral blood, Ki67^+^CD8^+^ cells peaked by day 17 in both PTs and AGMs, with a delayed increase in HLA-DR^+^CD8^+^ cells (Figure 1C, upper). Subsequently, immune activation (Ki67^+^) of circulating CD8^+^ T cells reached a plateau in PTs by day 28, whereas it progressively returned to baseline levels in AGMs by day 45+. Between days 10 and 45+, CD8^+^ T cell immune activation (Ki67^+^) was increased in the lymph node and bone marrow of PTs but not AGMs, but persisted in the colon of both species (Figure 1C, lower). Concordant with these results, histological examination of the lamina propria in the colon of both species showed an infiltration of Ki67^+^CD8^+^ T cells at day 45+ whereas, by this time, the density of Ki67^+^CD8^+^ T cells in the lymph node was only significantly increased in the PT (Figure S1C). Finally, sustained and generalized immune activation (Ki67^+^) was observed at necropsy (day 45+) in PBMCs, spleen, tonsil, bone marrow, thymus, axillary, iliac, and mesenteric lymph nodes, jejunum, and ileum of PTs but not AGMs (Figure 1D), and markers of inflammation (CXCL9/MIG) were increased in lymph nodes of PTs but not AGMs at early (day 10) as well as late (day 45+) time points after infection, while only slightly increased in colonic mucosal tissue of both species at later time points (Figure S1D).

These patterns of T cell activation closely approximated the kinetics of CD4^+^ T cell depletion in each organ studied. Thus, persistent T cell activation in the PT was associated with CD4^+^ T cell depletion in peripheral blood, lymph node, bone marrow, and colon (Figures 1E and S1E). In the AGM, the frequency and absolute number of circulating CD4^+^ T cells was lower than that of the PT prior to infection (see Text S1), but unchanged after infection (Figures 1E and S1E). The CD4^+^ T cell compartment of the SIV-infected AGM was also spared in the lymph node and bone marrow, but rapid and persistent CD4^+^ T cell depletion occurred in the colon, as has been reported previously [18] (Figures 1F and S1E). In addition, both PTs and AGMs were found to maintain a large population of memory CD4^+^ T cells, suggesting that differences in cytokine-producing T helper populations are not likely due to differential loss of this memory cell subset (Figure S1F and S1G, Text S2). Changes in the numbers of T cell subsets at various lymphoid sites in SIV infection have been described previously [18],[25],[27],[28],[29]. Although we observed a trend toward transient peripheral CD4+ T cell decline at day 10 in AGMs, this did not reach significance, presumably due to low baseline CD4^+^ T cell counts in these older AGMs. Of note, an abundant population of CD3^+^CD4^−^CD8^−^ “double negative” (CD3^+^DN) cells was observed in AGMs prior to SIV infection and remained unchanged after infection in the peripheral blood, lymph node, and colon (Figure S1E and S1F and Text S3).

Altogether, these findings indicate that both PTs and AGMs respond to SIV infection with acute and generalized T cell immune activation. Such activation continues unabated in the PT and is accompanied by CD4+ T cell depletion in multiple anatomic compartments. The AGM, by contrast, is able to dampen T cell immune activation in all compartments except the colon, wherein CD4+ T cell depletion occurs.

### CD4+ T cell depletion in the PT is accompanied by distinct inflammatory responses

**Figure 2:**
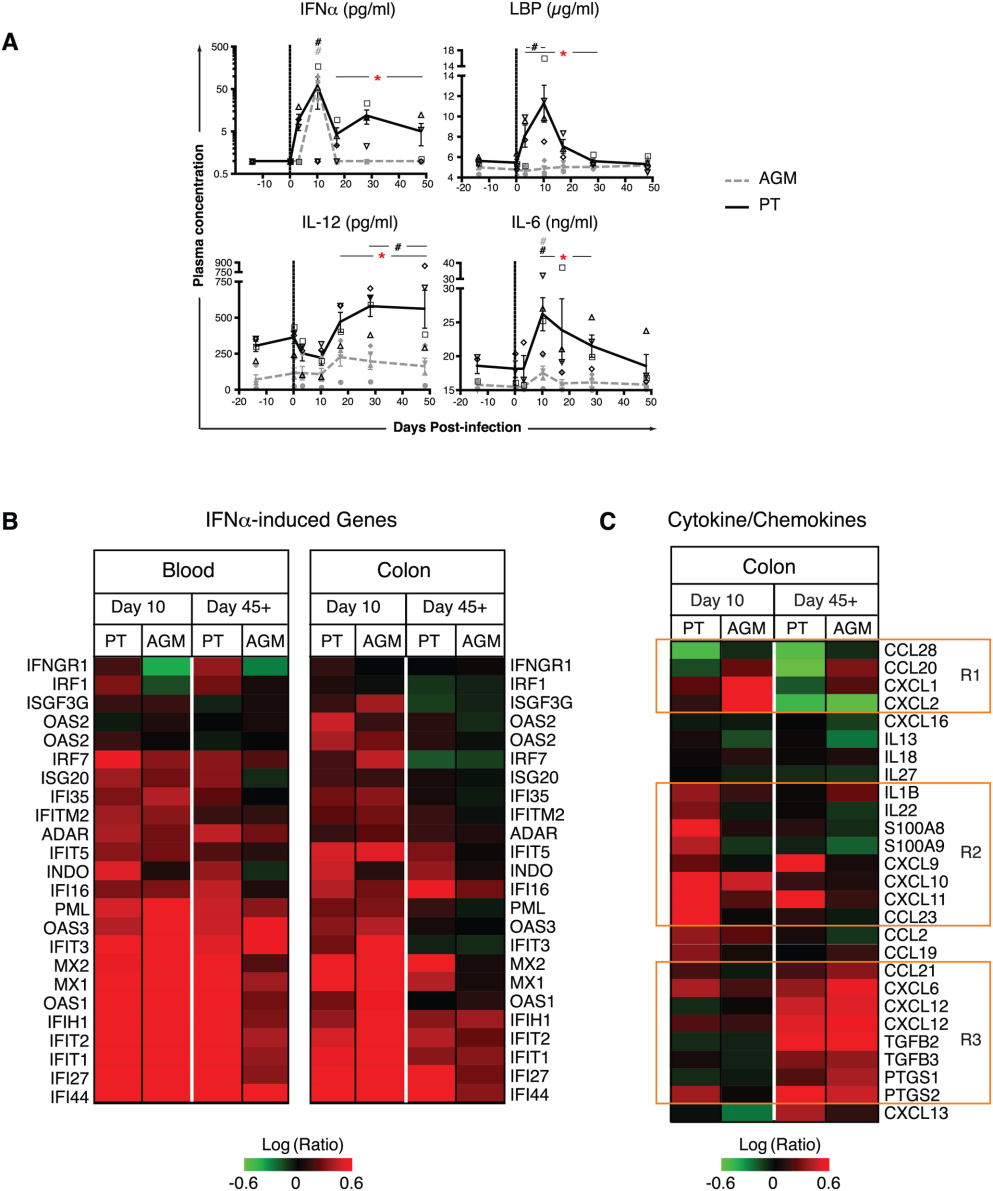
Differential production and transcription of cytokines, chemokines, and IFN-induced genes after SIV infection of PTs and AGMs. (A) Plasma levels of IFNα, LPS-binding protein (LBP), IL-12, and IL-6, as determined by ELISA. Refer to Figure 1 for a description of statistical analysis and symbols. (B and C) Microarray analysis of IFNα-induced genes (B) and cytokine/chemokine gene expression (C) in PTs (n = 4) and AGMs (n = 4) in blood and colon at days 10 and 45+. The data presented are gene expression changes calculated from technical replicate arrays (n = 2) and error-weighted averages for 4 animals (n = 8 arrays) at day 10 or day 45+ relative to baseline day −14 of each individual (see Materials and Methods, Table S1 and Protocol S1 for details). Rectangles R1, R2, and R3 (C) depict sets of cytokine and chemokine genes that are differentially regulated in AGMs and PTs over time, including those that are upregulated in AGMs and/or downregulated in PTs at day 10 (R1), upregulated in PTs but not (or downregulated) in AGMs at day 10 (R2), and upregulated both in PT and in AGMs at day 45+ (R3). Refer to Figure 1 for a description of statistical analysis and symbols.

To investigate whether the CD4+ T cell depletion in PTs correlated with differential changes in systemic inflammation and cytokine responses, we analyzed circulating cytokines and acute stress proteins in plasma. This analysis revealed high and sustained levels of IFNα and IL-12, as well as an acute elevation of lipopolysaccharide-binding protein (LBP) and of IL-6 in the peripheral blood of PTs but not of AGMs (Figure 2A).

We further analyzed the transcriptional profiles of IFNα-induced genes and of cytokines/chemokines by microarray in the peripheral blood and colon of PTs and AGMs at the peak of viremia (day 10) and at set point (days 45+) relative to baseline (day −14). In temporal concordance with the elevated levels of IFNα observed in the peripheral blood, a constellation of IFNα-induced transcripts was observed in the peripheral blood and colon of both PTs and AGMs at day 10 (Figure 2B). The levels of many of these returned to baseline by day 45+ in AGMs but not in PTs, in whom increased transcript levels from certain genes and gene families (e.g., PML, IFIs, OAS1, MX) persisted to day 45+. In other cases (e.g., IRF1 and INDO), the patterns of expression were different, with increased levels in PTs and decreased levels in AGMs at both early and late time points.

Transcriptional profiling of cytokines/chemokines in the colon of PTs and AGMs revealed additional differences in the response of these two species to SIV infection (Figure 2C). In PTs but not in AGMs, the peak of viremia (day 10) was associated with enhanced transcription of genes associated with acute inflammation and bacterial infection (e.g., IL-1β, IL-22, S100A8-9) [30], and with the chemotaxis of activated and differentiated Th1 and myeloid cells (e.g., CXCL9/MIG, CXCL11/I-TAC, CCL23/MPIF-1), while transcriptional levels of some genes associated with the chemotaxis of resting T cells and immature dendritic cells (e.g., CCL28/MEC, CCL20/LARC) were reduced (Figure 2C, R1–R2). By contrast, the transcription of CCL20/LARC and CXCL1-2/GRO was acutely elevated in AGMs but significantly and durably reduced in PTs, suggesting a change in Th17 environment with the recruitment of neutrophils (CXCL1-2), immature dendritic cells, and CCR6-expressing Th17 cells themselves (CCL20) [31],[32] (Figure 2C, R1). Both PTs and AGMs showed enhanced transcription of a number of genes in the colon at days 45+, in a pattern consistent with increased myelopoiesis and B lymphopoiesis (e.g., CXCL12), and with the establishment of an anti-inflammatory and immuno-protective response (e.g., TGFβ1I1, 2, and 3, PTGS1-2/COX1-2) (Figure 2C, R3).

In aggregate, these results reveal an acute inflammatory environment in PTs that is biased towards a Th1 response, more so than that which is found in AGMs. They also reinforce the conclusion that the AGM is able to dampen many gene functions associated with inflammation after the peak viral load (day 10), a point elaborated in a companion paper detailing the kinetics and anatomic location of gene expression changes in these species after SIV infection (Lederer et al.). We hypothesized that this dichotomy between the species might be due to the fact that pathogenic infection of the PT is driven by an increased frequency of pro-inflammatory Th17 cells [23],[24],[33] and/or a decreased frequency of anti-inflammatory Tregs [27],[34]. Further examination of this hypothesis provided unexpected results.

### IL-17-expressing Th17 cells are lost after SIV infection of the PT but not of the AGM

**Figure 3:**
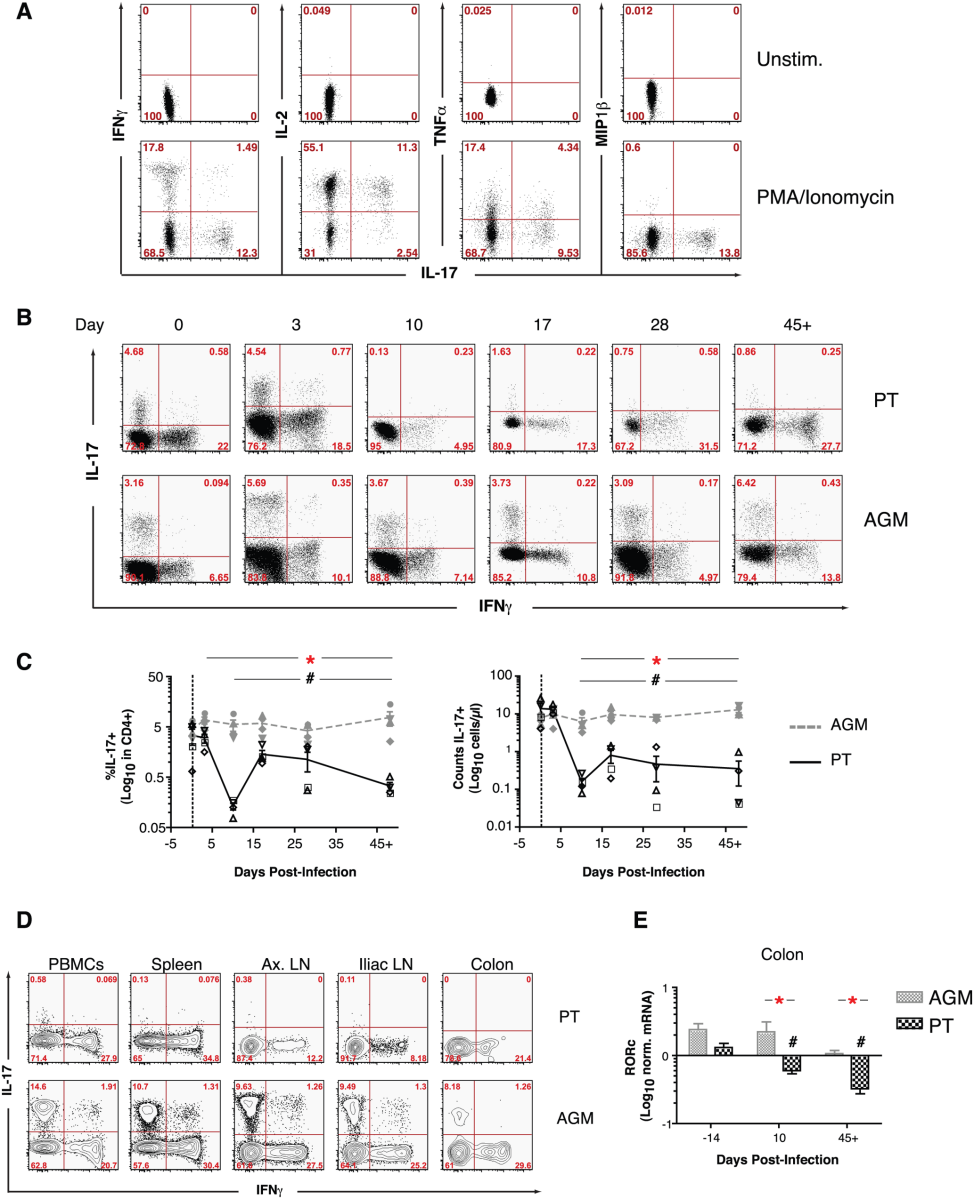
Acute SIVagm infection results in the loss of Th17 cells in PTs. (A) Multiparameter flow cytometric analysis of mock- or PMA/ionomycin-stimulated peripheral CD4^+^ T cells from a representative AGM (06004) at necropsy (day 45+), showing that IL-17-producing cells were CD4^+^ cells that produced IL-2 and TNFα, but only minimal amounts of IFNγ and no MIP1β. (B) Detection of IL-17 and IFNγ within CD3^+^CD4^+^ T cells as a function of time after infection in a representative PT (A99059) and AGM (06006). IL-17 was induced by PMA and ionomycin stimulation *in vitro*. (C) Time- and species-dependent changes in the frequency (left) and absolute number (right) of IL-17^+^CD4^+^ T cells in the peripheral blood. (D) Differential representation of IL-17^+^IFNγ^−^ cells in the peripheral blood, spleen, axillary and iliac lymph nodes, and colon of one PT (A99059) and one AGM (06004) at necropsy (day 45+). Of note, CD4^+^ T cells were substantially depleted in colon at this time point in both PTs and AGMs. (E) Real time PCR detection of changes in the relative proportion of Th17 cells in the colon, as measured by the expression levels of RORc. In (E), Mann-Whitney nonparametric test was used for log transformed values over time (#) (comparing each time point separately to baseline, e.g. day −14, in PTs or AGMs). Refer to Figure 1 for a description of statistical analysis and symbols in (C).

Th17 cells in blood and tissues of PTs and AGMs were identified by intracellular cytokine detection of the Th17-defining cytokine, IL-17A, in T cells stimulated with PMA and ionomycin. As has been found in human Th17 CD4^+^ T cells [31], CD4^+^ Th17 cells in the PT and AGM were of a lineage distinct from Th1, Th2, or Tregs cells (i.e., they produced only minimal amounts of IFNγ and no MIP1β, IL-4, IL-10, or TGFβ) (Figure 3A, and data not shown) and had a memory (CD45RA^−^CD27^+^) phenotype (data not shown). At the same time, a significant fraction of CD4^+^ Th17 cells produced IL-2 and TNFα (Figure 3A), and expressed the IL-2 receptor α chain (CD25) in the absence of FoxP3 (data not shown).

Prior to infection, Th17 CD4^+^ T cells were detected at the same frequency in AGMs and PTs (Figure 3B and 3C). After infection of PTs, the frequency (Figure 3B and 3C, left) and absolute number (Figure 3C, right) of circulating functional Th17 cells decreased dramatically; by contrast, Th17 cells were maintained at constant frequency and absolute number in the AGM (Figure 3B and 3C). At necropsy (day 45+), the frequency of functional Th17 cells was maintained at high levels in the spleen, axillary and iliac lymph nodes, and colon of AGMs but was substantially reduced in these tissues in PTs (Figure 3D). The depletion of Th17 cells in the lymph node and colon of PTs was also evident when mRNA levels of IL-17A or RORc [a specific lineage marker of Th17 cells [35]] were analyzed by RT-PCR (Figure 3E and data not shown).

### The loss of Th17 cells in PTs is selective

**Figure 4:**
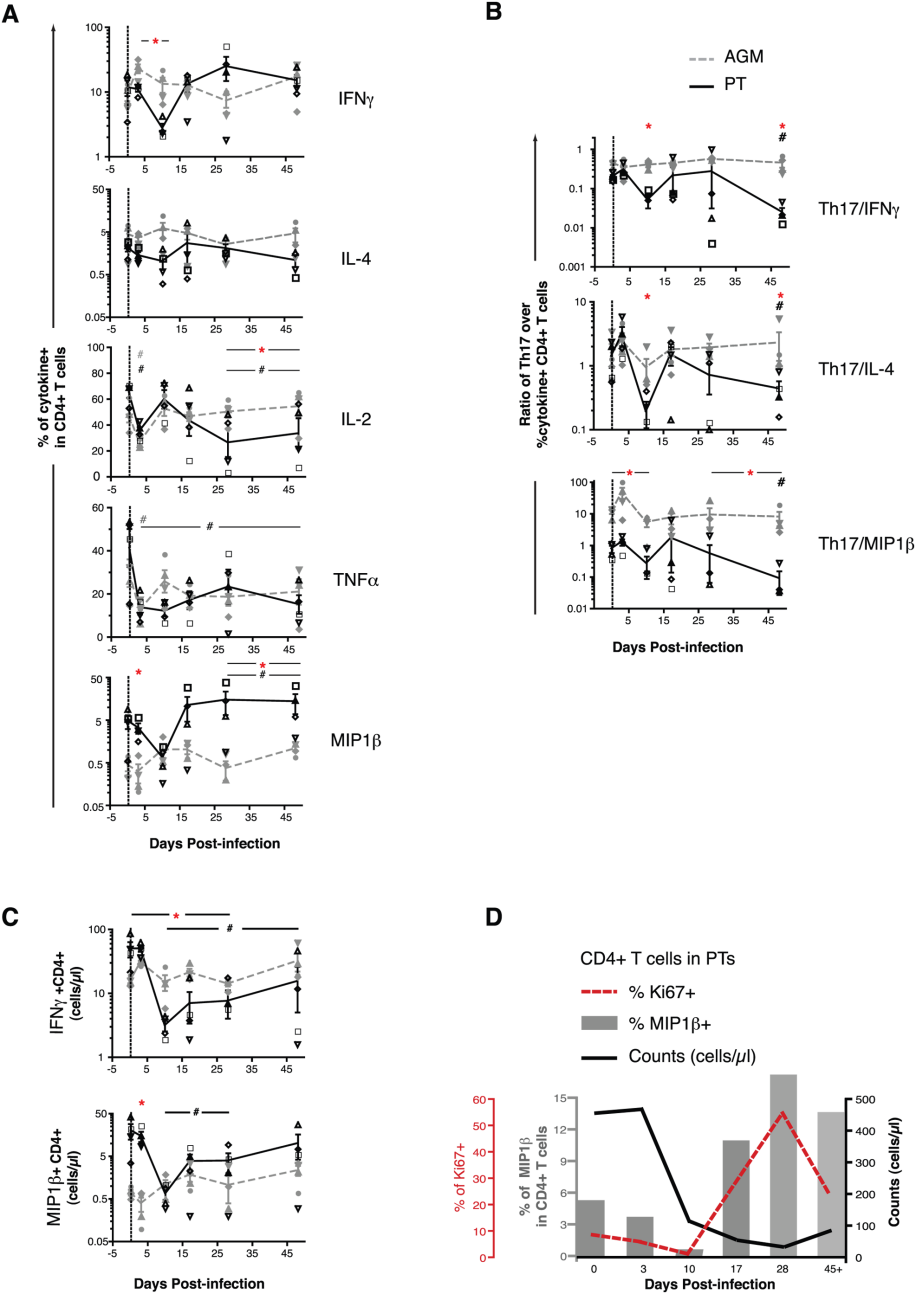
Selective depletion of Th17 cells in PTs. (A) Frequency of IFNγ, IL-4, IL-2, TNFα, or MIP1β cytokine-producing CD4^+^ T cells in PBMCs, detected after PMA and ionomycin stimulation *in vitro*. (B) Ratio of the fraction of Th17 cells over the fraction of CD4^+^ T cells producing IFNγ, IL-4, or MIP1β. (C) Cell counts of IFNγ and MIP1β cytokine-producing CD4^+^ T cells in PBMCs. (D) Frequency of CD4^+^ T cells expressing MIP1β or Ki67 related to the circulating CD4^+^ T cell count in PTs as a function of time after infection. In (B), Mann-Whitney nonparametric test was used for ratios over time (#) (comparing each time point separately to baseline, e.g. day 0, in PTs or AGMs). Refer to Figure 1 for a description of statistical analysis and symbols in other cases (A and C).

To understand whether the loss of Th17 cells in the PT was selective or simply due to generalized CD4^+^ T cell depletion, the frequency of cytokine-expressing Th1 cells (e.g., expressing MIP1β, IL-2, TNFα, and/or IFNγ) and Th2/Treg cells (e.g., expressing IL-4, IL-10, and/or TGFβ) was examined in each species. In both PTs and AGMs, acute SIV infection was associated with the maintenance of CD4^+^ T cells producing IFNγ or IL-4, and the loss of those producing TNFα (Figure 4A). By day 28, MIP1β-producing CD4^+^ T cells were enriched (p = 0.02) and IL-2 producing CD4^+^ T cells were depleted in PTs but not AGMs (p = 0.004) (Figure 4A). Depletion of IL-2 producing CD4^+^ T cells was due to the depletion of multifunctional IL-2^+^IL-17^+^(+/−TNFα^+^) Th17 cells and IL-2^+^IFNγ^+^ and/or TNFα^+^ Th1 cells (Figure S2A and Text S4 for the analysis of multifunctional cytokine profile, including antigen-specific responses). To determine whether there was selective depletion of Th17 cells over other Th populations (e.g., those producing IFNγ, IL-4, or MIP1β) we measured the ratio between Th17 cells and these other populations as a function of time post-infection. All of these ratios declined to significantly lower levels in PTs, but not in AGMs, by day45+ (Figure 4B). At this time, selective depletion of Th17 cells compared to all other cytokine-expressing populations was evident in PTs in blood and in all lymphoid tissues at necropsy (Figure S2B and S2C, and data not shown). In terms of absolute cell numbers, these changes in the PT were accompanied by an increased number of circulating Th1 MIP1β-expressing and monofunctional IFNγ-expressing CD4^+^ T cells in PTs, leading to partial recovery of Th1 cell counts over time (Figure 4C). As shown in the specific case of MIP1β (Figure 4D), a detailed kinetic analysis of these changes in blood indicated that they occurred after acute CD4^+^ T cell depletion and in parallel with the peak of the CD4^+^ proliferative (Ki67+) response in PTs. These changes point to *de novo* generation of Th1 cells and depletion of multifunctional IL-2 producing CD4^+^ T cell populations in PTs but not AGMs during acute SIV infection, and suggest that the generalized CD4^+^ T cell losses observed in the PT are accompanied by selective depletion of Th17 cells.

### FoxP3^+^ Tregs are sustained in the AGM and transiently lost in the PT

The frequency and number of FoxP3^+^CD4^+^ Tregs was determined in each species as a function of time post-infection. In each, these cells showed high levels of CD25, low levels of CD127 and of intracellular cytokines (e.g. IL-17, IL-2, IFNγ and TNFα) (Figure S3A and S3B, and data not shown), and *in vitro* suppressive activity comparable to that observed in the SIV-infected rhesus macaque [36] (Figure S3C and S3D).

**Figure 5:**
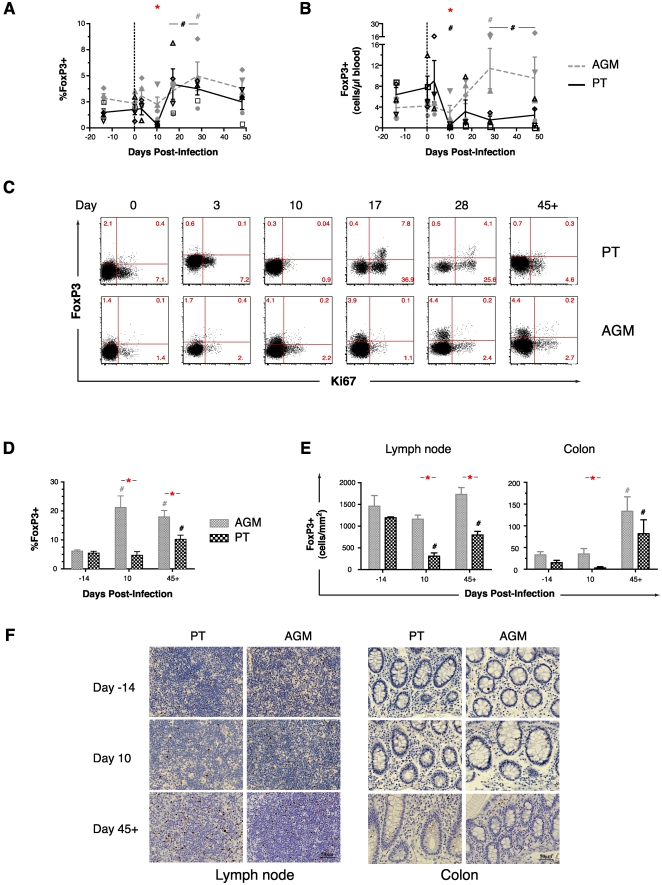
Differential dynamics of Tregs after acute SIV infection of PTs and AGMs. Frequency (A) and absolute number (B) of FoxP3^+^CD4^+^ T cells amongst total CD4^+^ T cells in PBMCs. (C) FACS detection of FoxP3 and Ki67 in peripheral blood CD4^+^ T cells as a function of time after infection in a representative PT (A99059) and AGM (06006). (D) Frequency of FoxP3^+^ cells in the lymph nodes both within CD4^+^ T cells. Quantitative analysis (E) and immunohistochemistry (F) of FoxP3-expressing cells within the paracortical T cell zone of lymph nodes (left) and in the lamina propria of colon biopsies (right). Refer to Figure 1 for a description of statistical analysis and symbols.

Between days 10–28 after SIV infection, the frequency of circulating FoxP3^+^CD4^+^ T cells increased in both species (Figure 5A, see also Figure S3E and S3F and Protocol S1 for details on the gating strategy of FoxP3^+^CD4^+^ T cells). Given the depletion of CD4^+^ T cells in PTs, however, high absolute numbers of such cells were found only in AGMs (Figure 5B). Notably, most FoxP3^+^CD4^+^ T cells on days 17 and 28 in PTs (but not AGMs) were activated (Ki67^+^) (Figure 5C), consistent with an induced phenotype in this species [34]. In lymph nodes, AGMs showed an early increase (day 10) in the frequency of FoxP3^+^CD4^+^, which was sustained to day 45+ (Figure 5D) and that was accompanied by a stable density of FoxP3^+^ cells (as measured by quantitative image analysis) (Figure 5E and 5F, left) and a stable level of FoxP3 transcripts (as measured by RT-PCR) (Figure S3G, upper). In lymph nodes from PTs, by contrast, the frequency of FoxP3^+^ cells was increased only at day 45+ (Figure 5D) and the density decreased by day 10, returning towards baseline by day 45+ (Figure 5E and 5F, left). Colonic tissue in both species revealed only a late (day 45+) increase in the density of FoxP3^+^ T cells (Figure 5E and 5F, right) and in the level of FoxP3 transcripts (Figure S3G, lower).

Thus, and contrary to our initial hypothesis, AGMs maintained a high number and frequency of Th17 cells in peripheral blood and lymphoid organs whereas PTs with progressive disease showed early, generalized, and selective depletion of Th17 cells. Concomitantly, AGMs responded to SIV infection with a rapid and durable systemic mobilization of FoxP3^+^CD4^+^ Tregs whereas, in PTs, such cells were induced transiently in the blood and only at later time points in lymph node and colonic tissue. We next investigated whether the acute changes that occur in these critical T cell subpopulations post-infection might predict and/or be associated with immune activation.

### Th17 depletion predicts systemic and sustained T cell immune activation

To investigate the impact of Th17 cells, viral load, and CD4 depletion on systemic immune activation, a mixed-effects analysis was performed with the frequency of Ki67^+^CD8^+^ T cells as the primary outcome. All post-infection measurements taken in both species at days 3, 10, 17, 28, and 45+ were included. The predictor variables (i.e., Th17 frequency, log_10_-transformed plasma viral load, and CD4 counts) were independent time-varying covariates. As expected, plasma viral load was not predictive whereas Th17 frequency and CD4^+^ T cell counts were both predictive of T cell immune activation (Ki67^+^CD8^+^) in univariate analysis, thereby emphasizing the relationship between immune activation and Th17 frequency and/or between immune activation and pathogenic outcome (CD4^+^ T cell depletion), independently of viral load in this model. Most importantly, in a multivariate mixed-effects analysis including the frequency of Th17 cells and CD4^+^ T cell counts, both variables were independent predictors of T cell immune activation (−1.82 lower CD8^+^Ki67% per each 1 percentage point higher of CD4^+^ Th17%, p = 0.004, and −0.036 lower CD8^+^Ki67% per each 1 percentage point higher CD4^+^ T cell counts, p = 0.005). In aggregate, these results demonstrate that the loss of Th17 cells (but not Th1, Th2 or Treg cells, data not shown) was a strong and independent predictor of increased systemic immune activation during acute SIVagm infection of AGMs and PTs.

### At set point, the loss of balance between Th17 and Tregs correlates with sustained systemic immune activation

**Figure 6:**
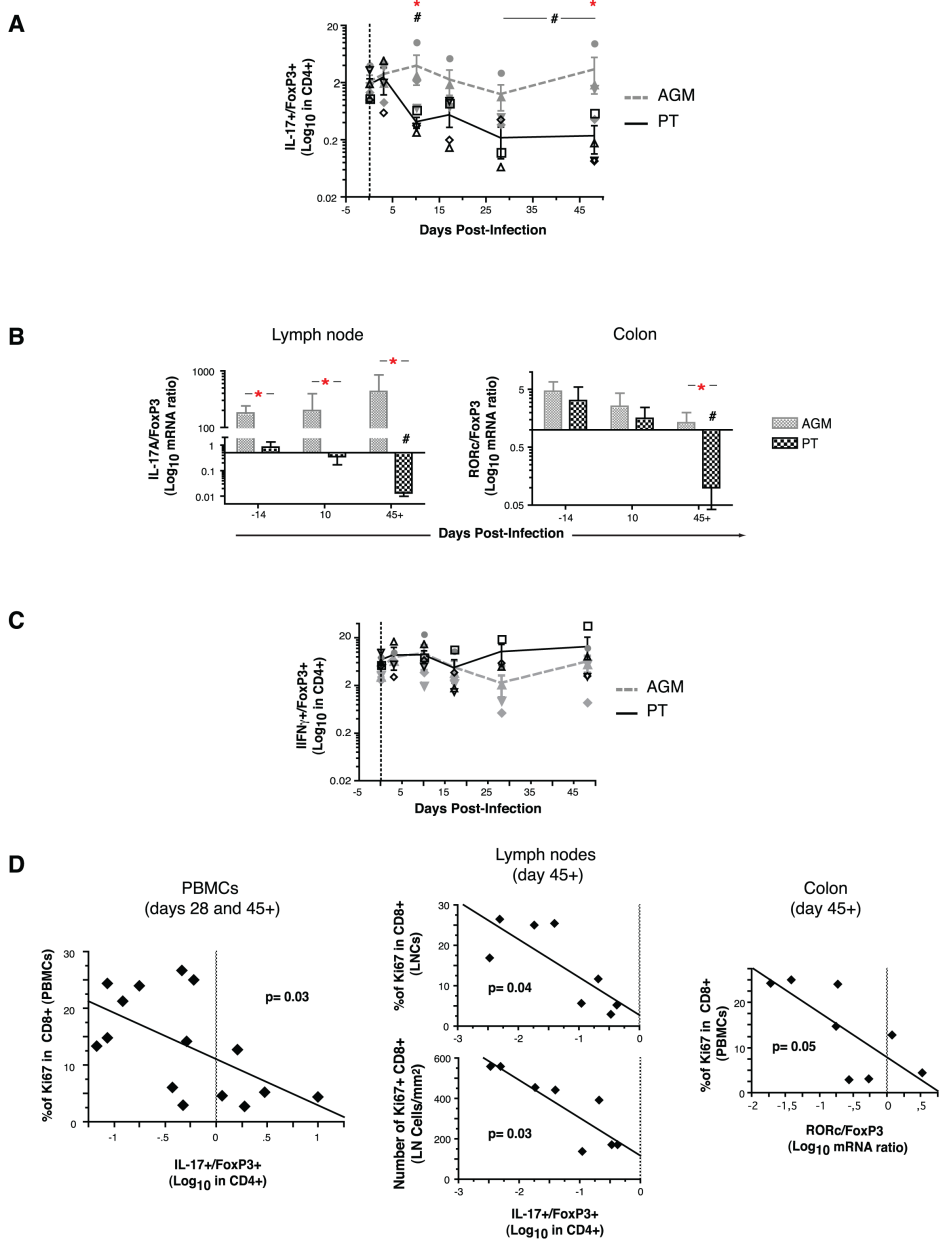
Loss of balance between Th17 and Treg cells correlates with pathogenic SIV infection and sustained immune activation after acute SIV infection of PTs and AGMs. Time- and species-dependent changes in the ratio of Th17/Treg for IL-17-producing CD4^+^ T cells and FoxP3^+^CD4^+^ T cells in the peripheral blood, as measured by flow cytometry (A) and by the fold change of the normalized mRNA expression levels of IL-17A (lymph node) or RORc (colon) over those of FoxP3 (B). (C) Ratio of Th1(IFNγ)/Treg as a function of time after infection. (D) Relationship between the Th17/Treg ratio in the peripheral blood, in lymph nodes and in colon to the degree of systemic immune activation, as measured by the fraction of Ki67^+^CD8^+^ T cells in blood (left and right) and in the lymph nodes (upper-middle) and by the density of Ki67^+^CD8^+^ cells in the paracortical T cell zone of lymph nodes (lower-middle) at viral and immunological set points (days 28 and 45+). Refer to Figure 1 and Figure 4 for a description of statistical analysis and symbols. Spearman's rank test was used to determine correlations between variables.

Given the counterposing biologic functions and reciprocal differentiation pathways of Th17 cells and Tregs [21],[22],[37],[38], we analyzed the relationship between these T cell subpopulations and signs of disease progression in PTs and AGMs. Combining the above observations on Th17 and Treg cells post-infection, AGMs were found to maintain high numbers of both subpopulations in the peripheral blood, resulting in a relatively stable ratio over time (Th17/Treg ratio ∼2.0 before and after infection) (Figure 6A). By contrast, SIV infection of PTs was associated with a rapid decrease in the ratio of circulating Th17 and Treg cells (Figure 6A), due both to a decreased frequency of Th17 cells (Figure 3B and 3C) and to an increased frequency of Tregs (Figure 5A) relative to baseline. By day 28, a 10-fold lower set point ratio (Th17/Treg ∼0.2) was reached, in a manner independent of the extent of CD4^+^ T cell depletion (Figure 6A). The relative loss of Th17 cells compared to Tregs in PTs was confirmed by RT-PCR analysis in the LNs and colon (Figure 6B, p≤0.03). This decline was specific to the Th17/Treg ratio, since the ratios of IFNγ-expressing Th1 cells and of IL-4-expressing Th2 cells to Tregs remained steady in each species during this same time frame (Figure 6C and data not shown). Most importantly, at the time when set points were established by day 28/45+, the Th17/Treg ratio in blood, lymph nodes and colon correlated negatively with sustained immune activation in blood and in lymph nodes at set point (p≤0.05) (Figure 6D), but not with colon immune activation (p = 0.3). A similar correlation was found when the Th17/Treg ratio was related to CD4+ T cell activation (%Ki67^+^) (p = 0.04, data not shown). These correlations were also specific to the Th17/Treg ratio, since ratios of Th17 to MIP1β- or IFNγ-expressing Th1 cells and ratios of Th17 to IL-4-expressing Th2 cells did not correlate with immune activation set points (data not shown). Thus, a balanced representation of Th17 and Treg cells was maintained upon nonpathogenic infection of the AGM. By contrast, SIV infection of the PT resulted in loss of Th17 cells and induction of Tregs, culminating in a lower Th17/Treg ratio, high levels of immune activation, and generalized CD4^+^ T cell depletion.

## Discussion

We report here a comprehensive virologic, histologic, immunologic, and gene expression analysis of acute infection in AGMs and PTs. Our results confirm and extend previous studies comparing the correlates of pathogenic versus nonpathogenic SIV infection in these two species [10],[18],[25],[27]. A number of novel features are also highlighted by the unique design of this experiment. First, sustained systemic immune activation (blood, lymph nodes, spleen) was only observed in PTs, while signs of T cell activation and inflammation were still observed in the colonic mucosa of both species. Second, an increase in the frequency of FoxP3^+^ Tregs was detected in the blood and in the lymph nodes after infection of both species, with an earlier and more dramatic increase in AGMs that correlated with limited or resolved systemic inflammation and immune activation. Third, functional Th17 cells were maintained or increased in infected AGMs but were rapidly lost during the course of progressive SIV disease in PTs. Finally, and combining these observations at the time when immunological and viral parameters reached steady state (day28/45+), a normal ratio of Th17/Treg cells (∼2.0) was associated with limited immune activation and no disease progression, whereas loss of this ratio was associated with pathology and none of the other ratio including Th17 to other Th1 and Th2 populations were found to correlate with immune activation setpoints. These findings demonstrate, in sum, that nonpathogenic infection in non-human primates is associated with a state of chronic inflammation that may be restricted to the colonic mucosa during acute infection. Concomitantly, signs of such inflammation were not observed in the peripheral blood and lymphoid tissues, wherein high Th17 cell numbers and a balanced representation of Th17 and Treg cells were maintained after acute infection. These studies suggest that loss of the Th17 to Treg balance during acute infection is related to and predictive of lentiviral disease progression.

A number of studies have shown that FoxP3^+^ T regulatory cells exert anti-inflammatory functions and control self-reactive T cells, including Th1, Th2, and possibly, Th17 cells [39],[40]. In the context of acute and chronic infectious diseases, the net outcome of these effects remains unclear [34]. In the context of HIV and SIV disease, Tregs might decrease chronic immune activation, thereby slowing disease progression [41]; conversely, they might inhibit anti-viral immune responses, thereby hastening disease progression [42],[43]. Discriminating between these two counterposing effects has been hampered by the difficulty of precisely identifying distinct subsets of FoxP3^+^ Tregs *in vivo*, e.g., induced versus natural Tregs [44], and their mechanism of suppression in controlling immune responses [45]. In this study, FoxP3^+^ T cells in PTs expressed an activated (Ki67^+^) phenotype and were increased at later time points following infection, consistent with the induction of an adaptive Treg response that exerted limited, if any, suppression of T cell activation in the periphery. Interestingly, a late increase in FoxP3^+^ cells in the lamina propria of both AGMs and PTs also correlated with increased expression of TGFβ2-3 and immune activation, suggesting that induced Tregs were equally generated in AGMs and PTs at the time of mucosal immune activation and that they did not exert a significant anti-inflammatory function in this organ. By contrast, FoxP3^+^ T cells in the blood and lymph nodes of AGMs were increased very early, were not activated, and were sustained at high levels directly after infection, consistent with the possibility that these cells may exert an anti-inflammatory effect involved in the resolution of systemic immune activation which, in turn, may limit viral replication and spread.

Th17 cells represent a subpopulation of activated (CD25^+^) and highly differentiated tissue-tropic CD4^+^ T cells associated with autoimmune disorders [23]. By contrast, Tregs may be activated but are often anergic and their function is by nature immunosuppressive [46]. From a teleological standpoint, one may speculate that the turnover of Th17 cell population is tightly controlled *in vivo* by mechanisms such as apoptosis and/or AICD pathways [47],[48], IDO-mediated tryptophan-deprivation [49], or galectin-1 signaling [50], mechanisms that might not pertain to the turnover of Tregs [51]. If true, Th17 cells might be more sensitive to cell death induced by viral (including bystander HIV-dependent GP120-CD4-signaling [52],[53],[54]) and/or host factors, compared to Tregs or Th1 (IFNγ-secreting) CD4+ T cells, thus accounting for their selective depletion in the context of pathogenic lentiviral infection.

The selective depletion of Th17 cells in PTs and their maintenance in AGMs was unexpected. These cells have been causally related to the immunopathology of multiple chronic inflammatory diseases, including allergy, autoimmune disorders, and allograft rejection [23], and could conceivably play a major role in the inflammatory state associated with progressive lentiviral disease. On the other hand, Th17 cells have also been found to enhance host defenses against microbial agents [33], thereby maintaining the integrity of the mucosal barrier against microbial infections [55],[56],[57],[58],[59]. Thus, the up-regulation of IL-1β, IL-22, and the anti-microbial peptides S100A8 and S100A9 in colons of PTs [30],[60], but not of AGMs, reveals a transcriptional signature suggestive of bacterial translocation and compromised colonic mucosal integrity [17],[30]. Thus, the detailed analysis of genomic changes in the colon of PTs (see companion paper from Lederer et al.) revealed an acute stress response in the NF-kB and TLR signaling pathways (and, in particular, TLR2), consistent with the loss of mucosal integrity and bacterial translocation in PTs but not in AGMs. Reciprocally, in the AGM, Th17 cells may not be “proinflammatory,” but instead capable of reducing immune activation in and/or to mucosal tissue, thereby decreasing systemic inflammation by preventing further microbial translocation and spread [17],[61]. This conclusion is supported by recent studies demonstrating that Th17 depletion in the ileal mucosa from chronically SIV-infected macaques correlated with increased systemic dissemination of *Salmonella typhimurium* [62], and that Th17 cells are preferentially depleted in the gastrointestinal tract of SIV-infected rhesus macaques [63] and of HIV-infected individuals, but not of SIV-infected sooty mangabeys [64].

Th17 cells share common chemokine receptors (CCR6, CCR4) and homing properties (CCL20) [31] with Tregs and are derived from a common progenitor cell [35],[38], the differentiation of which is dependent upon the stimulation of mucosal dendritic cells and macrophages by microbial, parasitic, or fungal products [65],[66],[67] and cytokines including IL-23, IL-1β and IL-6 [65],[68]. Our results showed that Th17 cells were selectively depleted over Tregs in the PT whereas the production of both populations was maintained upon acute infection of the AGM, possibly as a consequence of maintained balanced differentiation from a common progenitor [35],[38]. The maintenance of Th17 cells in the colon of AGMs but not PTs seems paradoxical, since both species experienced massive CD4+ T cell depletion, high viral loads, and late T cell immune activation (Ki67^+^CD8^+^ infiltration) in this compartment. However, AGMs were found to harbor a large population of CD3^+^CD4^−^CD8^−^ (CD3^+^DN) cells that included FoxP3- and IL-17-expressing cells that may serve to complement Treg and Th17 function in the colon in the absence of CD4^+^ T cells (data not shown). In addition, the preservation of Th17 cells in lymphoid tissues of AGMs, but not of PTs, may compensate for Th17 CD4^+^ T cell depletion in the colonic mucosa, by continuous recruitment and trafficking to the gut [69]. Despite similar levels of CD8^+^ T cell immune activation at day 45+ in the colon, the cytokine/chemokine “signature” of each species differed during early acute infection, suggesting recruitment of CXCR3-expressing Th1 cells (CXCL9) but not CCR6-expressing Th17 cells (CCL20) in PTs [31],[32] (Figure 2C, R1–R2). In addition, because IL-12 and Th-1 cytokines have been shown to inhibit Th17 lineage commitment [21], the observed skewed distribution toward Th1 cells and Th1-type cytokines and chemokines (e.g., CXCL9, IL-12, and IFNα) in PTs (Figure 2), but not in AGMs, may further impair subsequent Th17 lineage commitment, resulting in long-lasting depletion of the Th17 pool during chronic infection.

The observed relationship between Th17 cells and Tregs in the AGM prompts reconsideration of the role of these two cell populations during acute lentiviral infection. For instance, an increased representation of Tregs in the AGM after acute infection was not associated with diminished control of virus. Conversely, high levels of Th17 cells were not associated with enhanced immune activation and/or inflammation. Instead, Tregs present in the AGM may control immune activation in lymphoid organs and the effects of Th17 cells may serve to limit microbial translocation in the gut, thereby preventing the induction of systemic immune activation.

At this point in time, it is not feasible to show definitively that alteration of this balance (e.g., using specific monoclonal antibodies to modulate the Th17 or Treg populations in vivo) will have an impact on disease progression. However, we demonstrate that the rapid loss of Th17 cells and the loss of Th17/Treg balance in the PT is associated with multiple signs of progressive lentiviral disease: a rapid decrease of CD4 counts; sustained immune activation and apoptosis in CD4^+^ and CD8^+^ T cells of all lymphoid compartments; increased and sustained levels of pro-inflammatory cytokines and IFNα; and increased expression in situ and by microarray of genes previously associated with disease progression). By contrast, we also show in this study and in a companion paper (Lederer et al.) that AGMs responded to SIVagm-infection with a strong, systemic, and generalized IFNα response at the peak of SIV viral load which resolved to near baseline levels when viral setpoints were established. These data, in particular, indicate that the lack of pathogenicity of SIV infection of the AGM is not due to primary dysfunction in the IFNα signaling pathway, as suggested to be the case in SIV-infected sooty mangabeys [70].

We believe that the results of this study are important for three reasons. First, they suggest that a balanced representation of both Tregs and Th17 cells may play a unique and possibly determinative role in the setting of acute lentiviral infection. Secondly, the data reported herein underscore a concept likely important for our understanding of lentiviral pathogenesis: at the time of acute infection, a robust representation of Tregs (generally thought to suppress antiviral responses) and of Th17 cells (generally thought to be proinflammatory and, hence, to enhance viral replication and spread) might be beneficial to the host – not detrimental. Finally, this study prompts novel considerations for future thoughts about HIV vaccine development. Instead of focusing solely on the elaboration of a robust antiviral adaptive systemic immune response, it may also be beneficial to elicit responses that limit mucosal depletion of Th17 cells immediately after acute infection and that tilt the Th17/Treg balance towards that found in the AGM. At worst, such a vaccine approach might result in a viremic state which, like that of the AGM, is ultimately nonpathogenic. At best, it may lead to a Th17/Treg response that diminishes the level of immune activation, thereby slowing the pace of viral replication and spread.

## Materials and Methods

### Virus

The original SIVagm.sab92018 primary isolate[25] was kindly provided by Dr. O. Diop at the Pasteur Institute in Dakar, Senegal (see also Protocol S1 for more details).

### SIVagm infection of non-human primates and tissue collection

All animal and *in vitro* procedures were performed using standard protocols and according to guidelines approved by the University of Washington Environmental Health and Safety Committee, the Occupational Health Administration, the Primate Center Research Review Committee, and the Institutional Animal Care and Use Committee.

The four adult male AGMs (10 years old, weighing 6.0 kg, interquartile range 5.8–6.2) and four adult male PTs (11 years old, weighing 16 kg, IQR 15.3–17.0) included in this study were housed at the Washington National Primate Research Center (WaNPRC). At day 0, animals were inoculated intravenously with 600 50% tissue culture infectious doses (TCID_50_) of SIVagmSab92018 and followed until necropsy at day 45 or day 49 (subsequently noted as day 45+). Blood samples were collected at days −14, 0, 3, 10, 17, 28, and 45+. At days −14, 10, and 45+, bone marrow aspirates were obtained from the manubrium and biopsies were obtained from the colon and lymph nodes (axillary and inguinal) and samples of tissue were also collected at necropsy (day 45+) from iliac and mesenteric lymph nodes, tonsil, spleen, thymus, duodenum, jejunum, and ileum. After purification, PBMCs, LN and colon cells, or cells from other tissues, e.g., those obtained at necropsy (see Protocol S1 for more details) were counted and resuspended at 3–8×10^6^ cells/ml for subsequent phenotyping and functional analysis in R-10 medium [RPMI 1640 medium (Invitrogen, Carlsbad, CA) supplemented with 10% FCS (Hyclone, Logan, UT), 10 mM Hepes, 2 mM L-glutamine, 50 U/50 µg of penicillin/streptomycin per ml and 0.1 mM GIBCO MEM Non-Essential Amino Acid Solution (all from Invitrogen, Carlsbad, CA)] (see Protocol S1 for more details).

### RNA extraction

Viral RNA was purified from plasma using QIAmp Viral RNA mini-kits (Qiagen). Whole blood was processed for microarrays with the PreAnalytix PAXgene blood RNA system (Qiagen). For viral load and gene expression (qRT-PCR) analyses of PBMCs, total RNA was purified using the RNeasy mini-kit after cell lysis and homogenization using a QIAshredder (both from Qiagen). For microarray, gene qRT-PCR, and viral loads on tissues stored in RNAlater (e.g., colon and lymph node biopsies) or in OCT, (e.g., additional lymph node biopsies), tissues were homogenized using a Polytron PT2100 tissue grinder (Kinematica, Switzerland) and RNA was extracted into TRIZOL Reagent (Invitrogen). Genomic DNA was removed from total RNA from each biopsy and from PBMC samples used for real-time gene expression assays (qRT-PCR) and for viral loads using DNA-free kits (Ambion), and an RT-free control was added to all PCR reactions to rule out DNA contamination. RNA concentrations from whole blood, PBMCs, and tissue samples were quantified on an ND-1000 UV-Vis spectophotometer (Nanodrop, Wilmington, DE) and controlled for integrity and purity on a capillary electrophoresis system (Agilent 2100 Bioanalyzer; Agilent Technologies, Santa Clara, CA) and processed for microarray following standard procedures (see also Protocol S1 for more details).

### Viral load measurement

RNA viral loads in plasma and tissues were measured by real-time quantitative PCR (ABI PRISM 7700, Applied Biosystem Foster City, CA) in a one step RT-PCR reaction (HoTaq One Step-RT PCR Mix; Molecular Cloning Laboratories, South San Francisco, CA), using primers and probes as previously described[25]. Briefly, primers and probes used to measure viral load in plasma and tissues were: LTR - 5′ J15S (5′-CTC GGT GTT CTC TGG TAA G-3′), 3′ J15S (5′-CAA GAC TTT ATT GAG GCA AT-3′), and 15 s probe (5′-FAM-CGA ACA CCC AGG CTC AAG CTG G-TAMRA-3′) [all RNase-free and HPLC-purified products from Integrated DNA Technologies (Coralville, IA)]. Because SIVagm viral loads in PT macaques were found to be extremely high, all samples were run twice or more, independently (in duplicates), using different sources of RNA standards and positive controls: briefly, RNA standards were produced *in vitro* (SP6/T7 transcription kit, Roche, Applied Science, Indianapolis, IN) from the LTR template made out of RNA extracts of SIVagm.sab92018(#800) by RT-PCR (primers T7-LTR-4S (5′ - TAA TAC GAC TCA CTA TAG GGA GAA CTG GGC GGT ACT GGG AGT GGC TT - 3′) and LTR-2A (5′ - ACC TAA GGC AAG ACT TTA TTG AGG - 3′) from Integrated DNA Technologies, and subsequently purified (QIamp Viral RNA kit – Quiagen), DNA treated (DNAse, Ambion) and controlled for RNA weight and purity. All standards were compared to internal controls (RNA from plasma other analyzed samples) and external RNA standards (kindly provided by Dr. Michaela Muller-Trutwin, Pasteur Institute, Paris, France).

### Expression microarray and real-time RT-PCR

Gene expression on whole blood and colon biopsies at days 10 and 45+ was compared to the baseline time point at day −14 for each tissue and each animal. Equal quantities of total RNA were amplified with a Low RNA Input Fluorescent Linear Amplification kit (Agilent Technologies, Santa Clara, CA). Probe labeling and microarray slide hybridization were performed with custom rhesus macaque (*Macaca mulatta*) oligonucleotide microarrays containing 22,000 rhesus probes corresponding to approximately 18,000 unique rhesus genes (see also Protocol S1 for more details). Raw microarray image files were processed using Feature Extraction 8.1 software (Agilent Technologies) and entered into a custom-designed relational database (Expression Array Manager) and analyzed with Rosetta Resolver System 6.0 (Rosetta Biosoftware, Seattle, Washington) and Spotfire Decision Site for Functional Genomics 8.1 (Spotfire, Somerville, Massachusetts).

A selected list of two sets of genes, e.g. IFNα-induced genes and of cytokine and chemokine genes (see Protocol S1 for the detailed list of genes) was analyzed for significant fold change over baseline at day 10 and day 45+ in two groups, including all 4 AGMs or 4 PTs. From this selected list of genes, only those showing greater than 99% probability of being differentially expressed (p≤0.01) and an expression level change of 2-fold or greater in at least one of the error-weighted combines on either day 10 or day 45+ for colon or blood (IFNα-induced genes) or for colon alone (cytokine and chemokine genes), were included for transcriptional profiling, as presented in Figure 2B and 2C and Table S1. Primary data will be available in the public domain through Expression Array Manager at http://expression.viromics.washington.edu/, in accordance with proposed MIAME standards, while a more complete analysis of the microarray dataset, including lymph nodes, is presented in the companion paper (Lederer *et al.*).

For gene expression by real-time RT-PCR, 1 µg of total DNA-free RNA from PBMCs, lymph node, and colon per animal per time point was retrotranscribed (Omniscript Reverse Transcription kit, Qiagen) with random primers (Stratagene, La Jolla, CA) supplemented with RNasin Plus RNase inhibitor (Promega, Madison, WI). Real-time PCR was run in duplicate for the HPRT housekeeping gene and the target gene on the same 96-well optical plate (Applied Biosystems, Foster City, CA) in a 25 µl reaction volume containing 5 µl cDNA (1/10 of RT product) plus 25 µl Mastermix (Applied Biosystems) and run on a real-time quantitative thermocycler (ABI PRISM 7700) following manufacturer's instructions. Primer-probe sets from Applied Biosystems included IL-17A rhesus (Rh02621750_m1), RORC (Hs01076112_m1), FoxP3 (Hs00203958_m1) and HPRT (Hs99999909_m1). Results were analyzed with the SDS 7900 system software, version 2.1 (Applied Biosystems). Cytokine mRNA expression levels were calculated from normalized ΔCT values as compared to HPRT. All samples from days −14, 10, and 45+ were run on the same plate for each target genes.

### Cell counts, phenotype, and functional analysis by flow cytometry

Panels of antibodies used for multiparameter flow cytometry are listed in Table S2 (including clone, manufacturer, and dilution). FACS analysis was performed on one or two four-laser BD LSR-II flow cytometers using High Throughput System (HTS) plate readers (BD Biosciences) and data were analyzed using FlowJo software v6-8 (Treestar, Ashland, OR).

Complete blood counts (CBC) were determined at the WaNPRC Clinical Lab in Seattle as well as by absolute counting on 50 µl whole blood, using Trucount absolute counting tubes (BD Biosciences, San Jose, CA). Cells counts in bone marrow were determined on Trucount tubes only, using 10 µl of bone marrow suspension. Phenotyping was performed by cell surface staining and, when necessary, by intracellular staining (Fix/Perm and Perm/wash buffers, BD or FoxP3 buffer set; Biolegend, San Diego, CA), following manufacturer's instructions (Table S2). Multifunctional cytokine assays were performed *in vitro* after no stimulation or after stimulation of 5–8×10^5^ cells with 5 µg/ml of SIVagm.sab92018 peptide pools (see Protocol S1 for details) or polyclonal activators (e.g., concanavilin A, 10 µg/ml; and PMA, 10 ng/ml; and ionomycin, 1 µg/ml) for 8–12 hours.

FACS data were analyzed using FlowJo software with standard gating strategies and then transferred into analysis and graphic software including Excel (Windows), StatView (Abacus Concepts, Berkeley, CA), SPICE (kindly provided by Dr. M. Roederer of the Vaccine Research Center, NIAID/NIH, Bethesda, MD), and Graphpad Prism4 (San Diego, CA). Multifunctional cytokine analysis was performed after stringent gating of each cytokine positive population and subsequent Boolean gating (FlowJo). All graphic data were represented on an absolute scale representing the frequency of the combination of cytokines secreted over time in CD4^+^ or CD8^+^ T cell populations (see Protocol S1 for more details).

### Histology, immunohistochemistry, and quantitative image analysis

H&E stained sections of lymph nodes and colon from paraffin-embedded biopsies and necropsy samples were examined by semi-quantitative histopathology analysis. For immunohistochemistry, all slides were stained using the Dako Autostainer (Dako Inc., Carpenteria, CA) and primary antibodies including biotin-labeled goat anti-MIG serum (R&D systems Minneapolis, MN), polyclonal anti-FoxP3 rabbit serum (Abcam, Cambridge, MA), polyclonal anti-CD8 rabbit serum (DBS, Pleasanton, CA), and polyclonal anti-Ki67 rabbit serum (Lab Vision, Fremont, CA). Tris-buffered saline (TBS) with Tween-20 (Dako Inc., Carpenteria CA) was used for all washes and as a diluent for antibody (Dako Inc., Carpenteria CA). Prior to staining, slides were subjected to an antigen retrieval step consisting of incubation in AR10 (Biogenex Inc, San Ramon, CA) for 2 min at 123°C in the Digital Decloaking Chamber (Biocare Medical Inc., Concord, CA), followed by cooling to 90°C before rinsing in running water and a final buffer rinse. Non-specific binding sites were blocked with a 30-minute incubation in DAKO protein block (Dako, Inc., Carpinteria CA) between the first primary antibody incubation and detection reagents, and the addition of second primary antibody and detection reagents. Primary antibodies were replaced by normal rabbit IgG (Zymed Inc., South San Francisco, CA) and included with each staining series as the negative control. Avidin-biotin complex (ABC complex) method and EnVision system (Dako, Inc., Carpinteria CA) were used as detection systems. DAB and Vector SG (Vector, Burlingame, CA) were used as chromogens.

Slides were visualized with a Zeiss Axioplan 2 microscope (Carl Zeiss Inc, Thornwood, NY). Digital images were captured and analyzed using a Zeiss Axiocam System and Openlab software (Inprovision Inc., Waltham, MA). One section per tissue with representative histomorphologic components (cortex, paracortex, follicles, medulla for LN; and epithelium, lamina propria, and muscularis mucosa for colon) was analyzed. Five high-power (40×) microscope fields of the T cell rich zone (paracortex) per LN section and five high-power fields of lamina propria from colon sections were randomly chosen and captured digitally with the system described above. Each captured field includes an area of approximately 0.09 mm^2^. Individual positive cells in the five captured high-power microscope fields of the immunohistochemical tissue sections were counted manually by a single observer. The numbers of positive cells are presented as cells per square millimeter.

### Measurement of plasma factors by ELISA

Plasma was stored at −80 C prior to analysis. Cytokine and lipopolysaccharide-binding protein (LBP) were measured using ELISA kits for IL-6 (Cytokine Bead Array for on–human primates; BD Biosciences, San Jose, CA), IL-12 (monkey) (U-Cytech CM, Utrecht, The Netherlands), IFNα (human) (PBL Biomedical Laboratories, Piscataway, NJ), and LBP (HK315) (Hycult Biotechnology, Uden, The Netherlands).

### Statistical analysis

Intracellular cytokine responses were analyzed using primarily Wilcoxon-Rank test, supplemented with Student t-test, as part of the SPICE platform comparing results at any time point to a day 0 reference. The Mann-Whitney U test was used for 2-group comparison between AGMs and PTs for each time point, whereas ANOVA was used to test differences over time compared to baseline for each group. For ratios and values expressed on log-transformed values (Th17/Treg, Th1/Treg, RORc/FoxP3), both ANOVA and Mann-Whitney U test were run to test differences over time. Mixed-effects longitudinal statistical models were used to test the impact of CD4^+^ T cell subsets (Th17, Treg, Th1), ratio (Th17/Treg), blood CD4 counts or CD4 loss, as well as viral load, on CD8+ T cell activation levels (%Ki67) during the infection period (day3 and thereafter). These models were run in the SAS System 9.2 and specified random effects for time and the individual. Spearman's rank correlation was used to determine correlations between variables. *P* values<0.05 were considered statistically significant.

## Supporting Information

Figure S1 Plasma viral load, T cell activation, and CD4^+^ and CD8^+^ T cell changes after SIV infection of PTs and AGMs. (A) Plasma viral load measured before and after inoculation of 4 PTs and 4 AGMs with the SIVagm isolate, SIVagm.sab92018 (600 TCID_50_). (B) Flow histograms showing the distribution of Ki67 expression in peripheral blood CD4^+^ T cells in a representative PT (J96082) and AGM (04005) before and after infection. (C–D) Immunohistochemistry (left) and quantitative image analysis (right) of (C) Ki67^+^CD8^+^ T cells (blue- and brown-colored) and (D) CXCL9/MIG-expressing (brown-colored) in the paracortical T cell zone (lymph node) and in the lamina propria (colon). (E) Flow cytograms showing the distribution of CD4^+^ and CD8^+^ T cells before and after infection of a representative PT (J96082) and AGM (06005), in peripheral blood (upper), lymph node (lower left), and colon (lower right). (F) Flow cytograms showing the distribution of naïve (CD45RA^+^CD27^+^), memory (CD45RA^−^CD27^+^), effector (CD45RA^−^CD27^−^), and terminally differentiated effector (CD45RA^+^CD27^−^) subpopulations among CD8^+^, CD4^+^ and CD3^+^DN T cells in one representative PT (J96082) and one representative AGM (06005). (G) Changes in T cell subpopulations after acute infection (see the legend to the left), depicted as pie-chart representations of the average mean frequencies of each of these subpopulations in PTs and AGMs, as a function of time post-infection in the peripheral blood (middle) and lymph nodes (right) in CD4^+^ PBMCs (upper) or CD8^+^ PBMCs (lower). Data are presented as means+/−SEM. Statistical analysis: *P* values were obtained on a per group basis (*) using the Mann-Whitney nonparametric test (when comparing PTs to AGMs at a given time point) or over time (#) by ANOVA (linear scale) or Mann-Whitney (for log transformed values and ratios) (comparing each time point separately to baseline, e.g. day −14 and day 0, in PTs or AGMs). Statistical analysis of subset cell composition (pie chart) was obtained by permutation analysis comparing each time point to baseline (day −14 and day 0) using SPICE software. When the *p* value is not indicated on the graph, “*” or “#,” indicate that *p*<0.05 for each test, respectively. NS means “not statistically significant.”
(6.86 MB PDF)

Figure S2 Changes in the frequency of multifunctional CD4^+^ and CD8^+^ T cells as a function of time post-infection in PTs and AGMs. (A) The frequency of multifunctional CD4^+^ T cells producing IL-2, TNFα, IFNγ, IL-17, and/or MIP1β was assessed by 10-color flow cytometry, analyzing cells secreting all 32 possible combinations of 1, 2, 3, 4 and/or 5 cytokines, with Boolean gating (FlowJo platform) and SPICE software (M. Roederer; described in Protocol S1). Rectangles R1, R2, and R3 highlight changes in the frequency of certain subpopulations that occurred over time primarily in the PT but not the AGM, whereas rectangle R4 highlights changes that occurred primarily in the AGM. Rectangles A, B, and C highlight IL-17^+^ subpopulations that also express IL-2 and TNFα (A), IL-2 alone (B), or no other cytokine from this list (C). Individual animals are represented by dots, means by dashed lines, and interquartile ranges by bars. *P* values (<0.05) were obtained using the Student's t-test (+) or Wilcoxon-Rank test (#), comparing mean values to the baseline values (day 0) for each species, as provided by SPICE software. (B) FACS detection of IL-17 (x-axis) and IL-2, TNFα, IFNγ, or MIP1β (y-axis) within CD3^+^CD4^+^ T cells over time in a representative PT (A99073) and AGM (06004) after PMA and ionomycin stimulation *in vitro*. (C) Differential representation of IL-17 (x-axis) and IL-2, TNFα, IFNγ, or MIP1β (y-axis) within CD3^+^CD4^+^ T cells in the peripheral blood, spleen and mesenteric lymph nodes of one representative PT (A99028) and one AGM (06005) at necropsy (day 45+).
(1.31 MB PDF)

Figure S3 FoxP3^+^ T cell populations in the PT and AGM, pre- and post-infection. (A) FoxP3 and CD127 (IL-7 receptor) expression on CD4^+^ T cells in PBMCs [from a chronically SIVagm.sab92018-infected AGM (18 months post-infection)]. (B) CD25-enriched (“CD25^+^ Pos. Selection”) fractions using anti-CD25 magnetic beads, stimulated *in vitro*, and then analyzed for expression of IL-17, IL-2 or IFNγ, and FoxP3. (C–D) Suppression of cell division by total CD25^+^ Tregs from PBMCs [from chronically SIVagm.sab92018-infected AGMs (18 months post-infection)] on CFSE labeled CD25^−^ PBMCs (Responder) gated on CD4+ T cells or CD8+ T cells with or without Treg add-back at a ratio of 1∶4 with responder cells (C-left). The same donor was tested with a dynamic range of Treg over responding cells with the frequency of undivided fraction indicated in CD4^+^ T cells or CD8^+^ T cells (C-right), showing augmented frequency of undivided cells as Treg/responder ratio increased. The same results were obtained when proliferation was analyzed using the FlowJo proliferation platform (D) from another AGM [% of Divided cells, Division index and Proliferation index], with data points indicating the % divided, proliferation index or division index achieved by CFSE-labeled cells in each sample as a percentage of the maximal value obtained in the CD25-depleted population with no add-back. (E. F) Simultaneous FoxP3 and Ki67 expression in CD4^+^ and DN T cells in the peripheral blood (upper) and axillary lymph node (lower) of a representative PT (A99059) (E) and AGM (06004) (at day 45+) (F). Also shown in each panel is the gating strategy for singlets, lymphocytes and CD3^+^ T cells. (G) FoxP3 mRNA levels in lymph node (upper) and colon (lower), as measured by real-time RT-PCR for FoxP3, with *P* values indicated in the PT lymph node and colon compared to baseline.
(5.66 MB PDF)

Protocol S1 (0.10 MB DOC)

Table S1 IFNα-induced and cytokine/chemokine transcripts analyzed in the arrays of Figure 2, B and C (see Protocol S1 for more details).
(0.21 MB PDF)

Table S2 Antibody panels for multiparameter flow cytometry. BD: BD Bioscience, San Jose, CA; Coulter: Beckman Coulter, Inc. Fullerton, CA, eBiosciences: eBiosciences, San Diego, CA; Biolegend: Biolegend, San Diego, CA, R&D: R&D Systems, Inc, Minneapolis, MN; Cedarlane: Cedarlane Laboratories Ltd. Burlington, NC; NPRR: National Institutes of Health (NIH) Nonhuman Primate Reagent Resource (NPRR) from K. Reimann (Harvard University, Cambridge, MA). CD38-FITC (OKT-10) is kind gift from R. Reyes, UCSF, San Francisco, CA.
(0.34 MB PDF)

Text S1 This text provides information related to the low frequency and absolute number of CD4^+^ T cells in the AGMs, as presented in Figures 1E and S1E.
(0.05 MB DOC)

Text S2 This text adds information related to T cell subpopulations as presented in Figures S1F and S1G.
(0.04 MB DOC)

Text S3 This text provides information related to the characterization of CD3^+^ CD4^−^CD8^−^ double negative cells (DN) as presented in Figures S1E and S1F.
(0.05 MB DOC)

Text S4 This text provides information related to Figure 4, A and B and Figure S2 related to the dynamic of Th1 and Th2/Treg CD4^+^ T cell populations as well as to SIV antigen-specific CD4^+^ and CD8^+^ T cell responses.
(0.06 MB DOC)
