# Supplementary figures and images for: Critical Loss of the Balance between Th17 and T Regulatory Cell Populations in Pathogenic SIV Infection
Source: PLoS Pathog. 2009 Feb 13;5(2):e1000295. doi: 10.1371/journal.ppat.1000295 (PMC2635016; doi:10.1371/journal.ppat.1000295)

**A**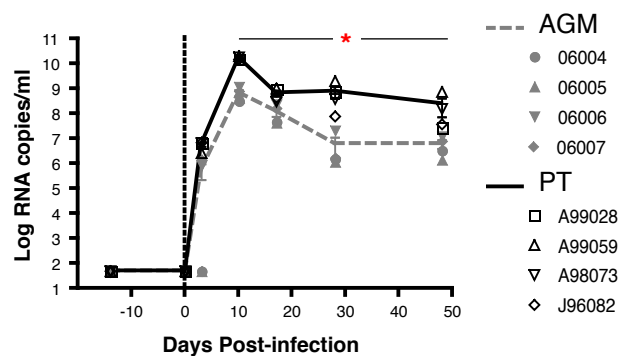**B**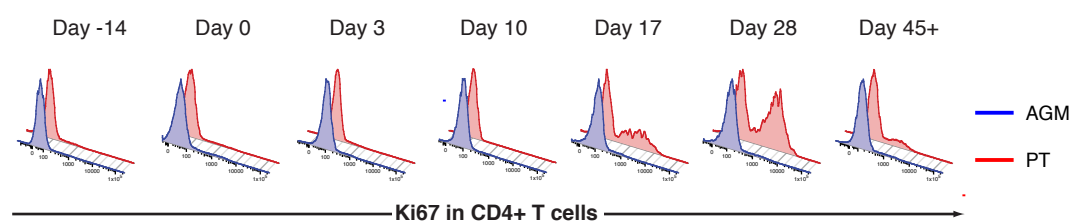**C**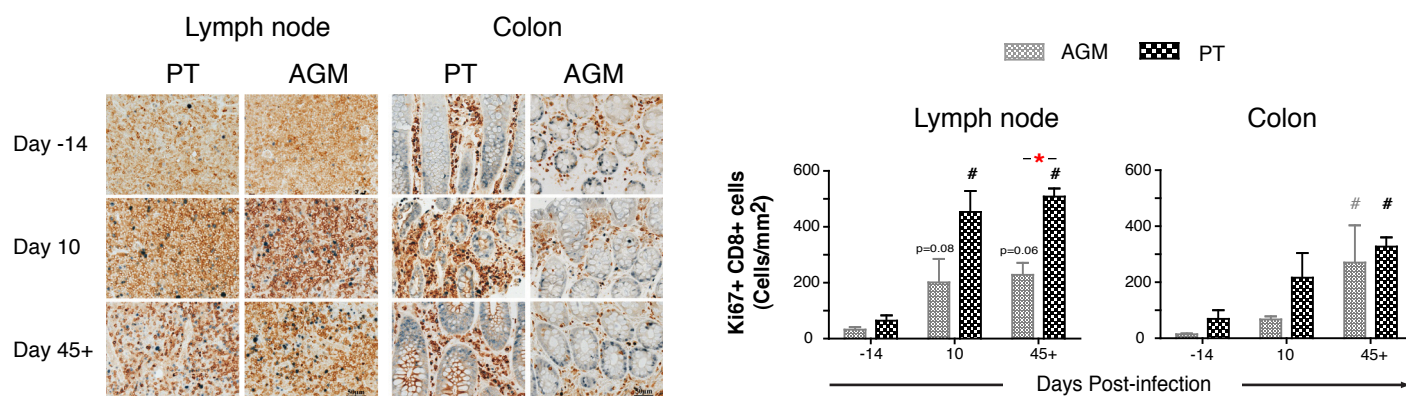**D**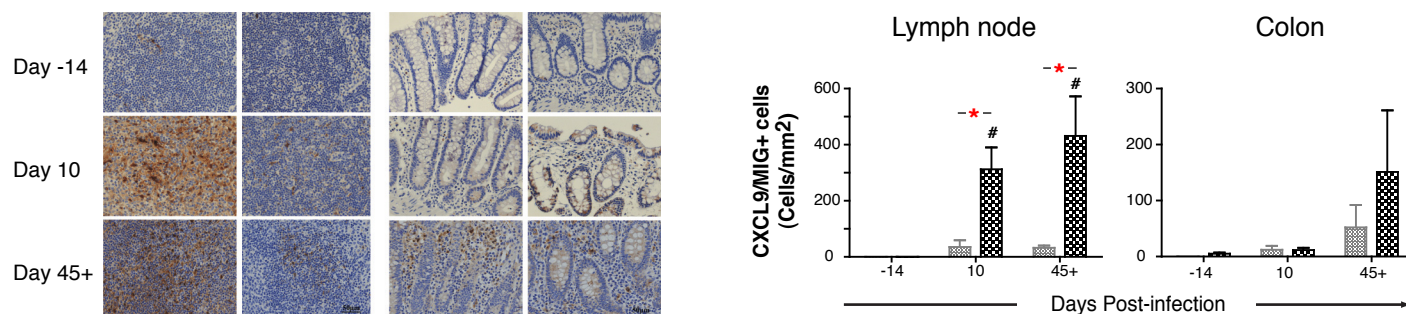

E

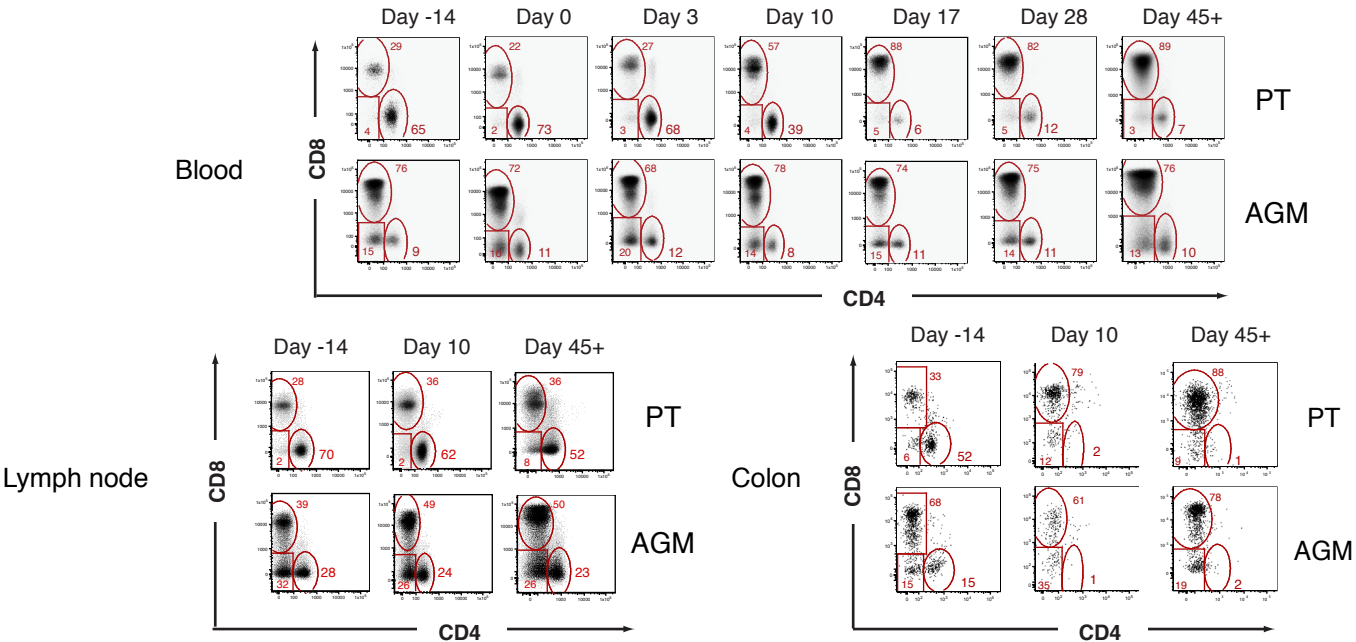

F

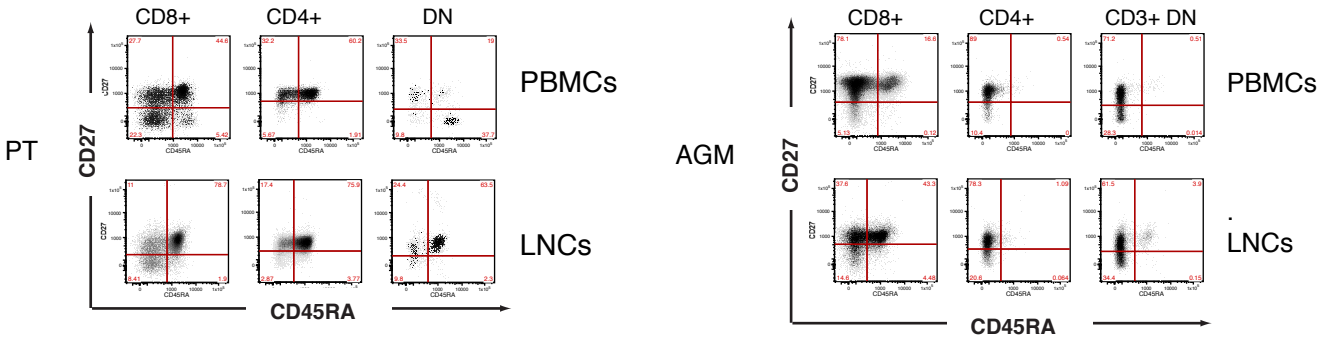

G

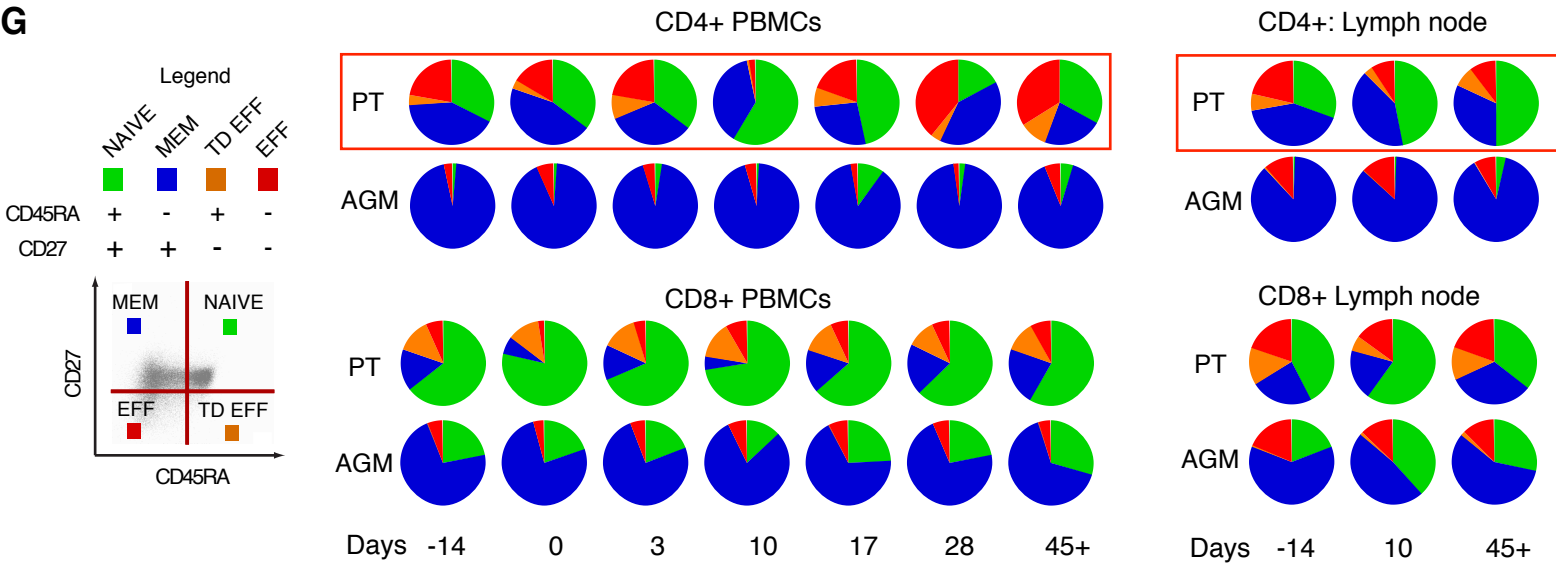

Supplement: Figure S1 — Plasma viral load, T cell activation, and CD4+ and CD8+ T cell changes after SIV infection of PTs and AGMs. (A) Plasma viral load measured before and after inoculation of 4 PTs and 4 AGMs with the SIVagm isolate, SIVagm.sab92018 (600 TCID50). (B) Flow histograms showing the distribution of Ki67 expression in peripheral blood CD4+ T cells in a representative PT (J96082) and AGM (04005) before and after infection. (C–D) Immunohistochemistry (left) and quantitative image analysis (right) of (C) Ki67+CD8+ T cells (blue- and brown-colored) and (D) CXCL9/MIG-expressing (brown-colored) in the paracortical T cell zone (lymph node) and in the lamina propria (colon). (E) Flow cytograms showing the distribution of CD4+ and CD8+ T cells before and after infection of a representative PT (J96082) and AGM (06005), in peripheral blood (upper), lymph node (lower left), and colon (lower right). (F) Flow cytograms showing the distribution of naïve (CD45RA+CD27+), memory (CD45RA−CD27+), effector (CD45RA−CD27−), and terminally differentiated effector (CD45RA+CD27−) subpopulations among CD8+, CD4+ and CD3+DN T cells in one representative PT (J96082) and one representative AGM (06005). (G) Changes in T cell subpopulations after acute infection (see the legend to the left), depicted as pie-chart representations of the average mean frequencies of each of these subpopulations in PTs and AGMs, as a function of time post-infection in the peripheral blood (middle) and lymph nodes (right) in CD4+ PBMCs (upper) or CD8+ PBMCs (lower). Data are presented as means+/−SEM. Statistical analysis: P values were obtained on a per group basis (*) using the Mann-Whitney nonparametric test (when comparing PTs to AGMs at a given time point) or over time (#) by ANOVA (linear scale) or Mann-Whitney (for log transformed values and ratios) (comparing each time point separately to baseline, e.g. day −14 and day 0, in PTs or AGMs). Statistical analysis of subset cell composition (pie chart) was obtained by permutat [file ppat.1000295.s001.pdf]

**A**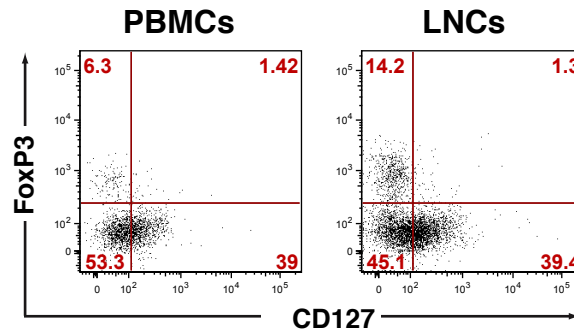**B**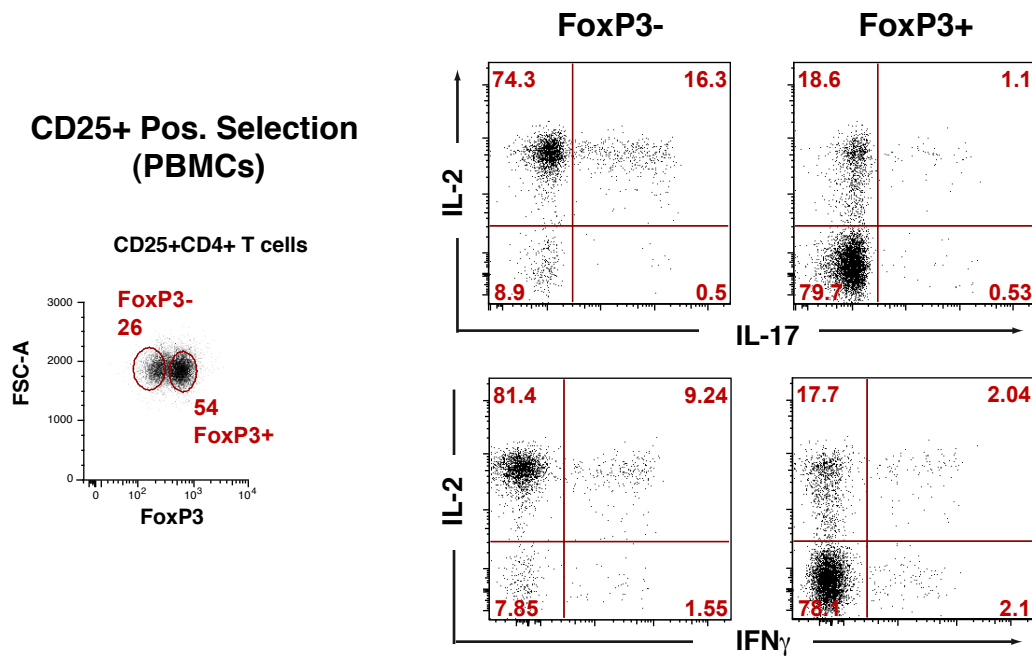

C

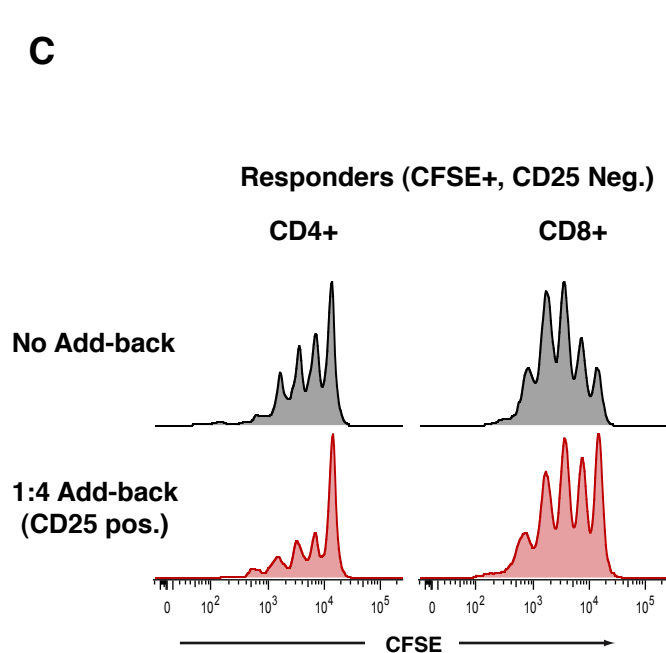

Treg/Responders

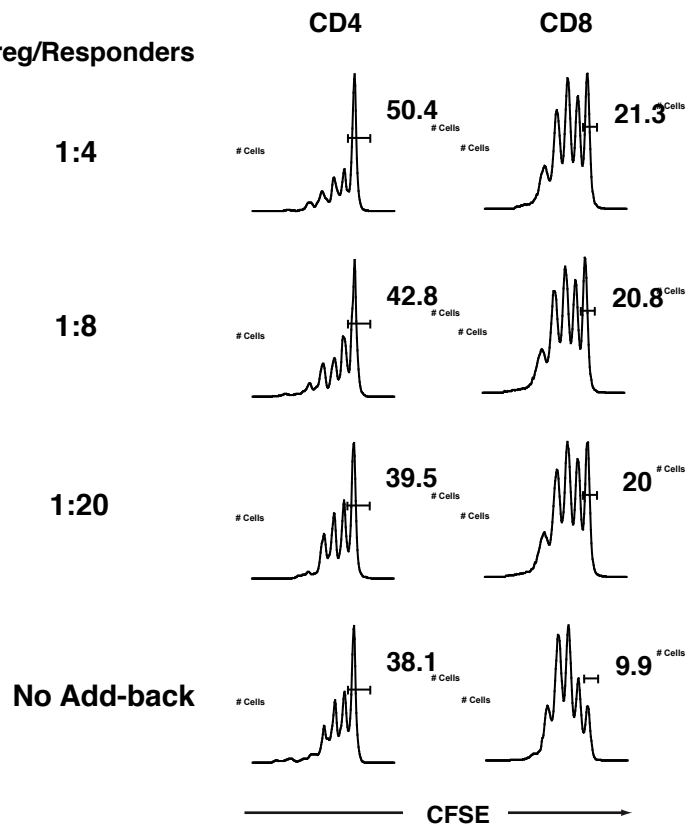

D

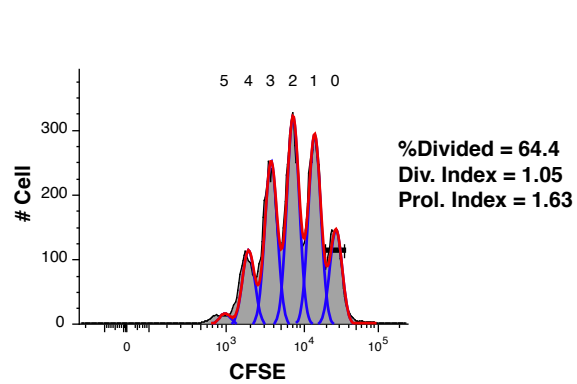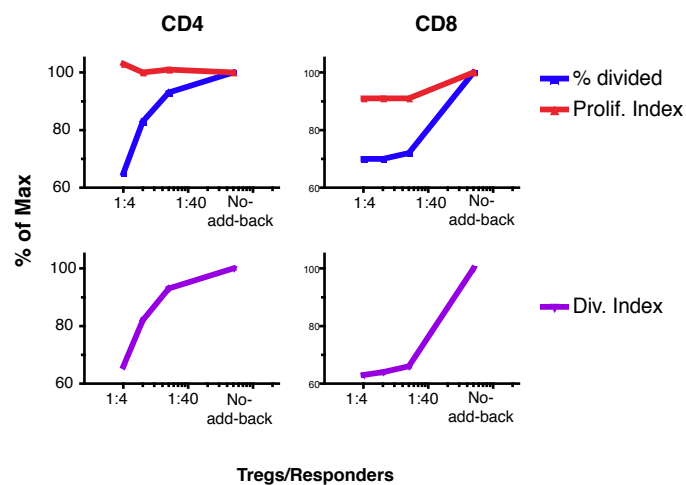

PT

PBMCs

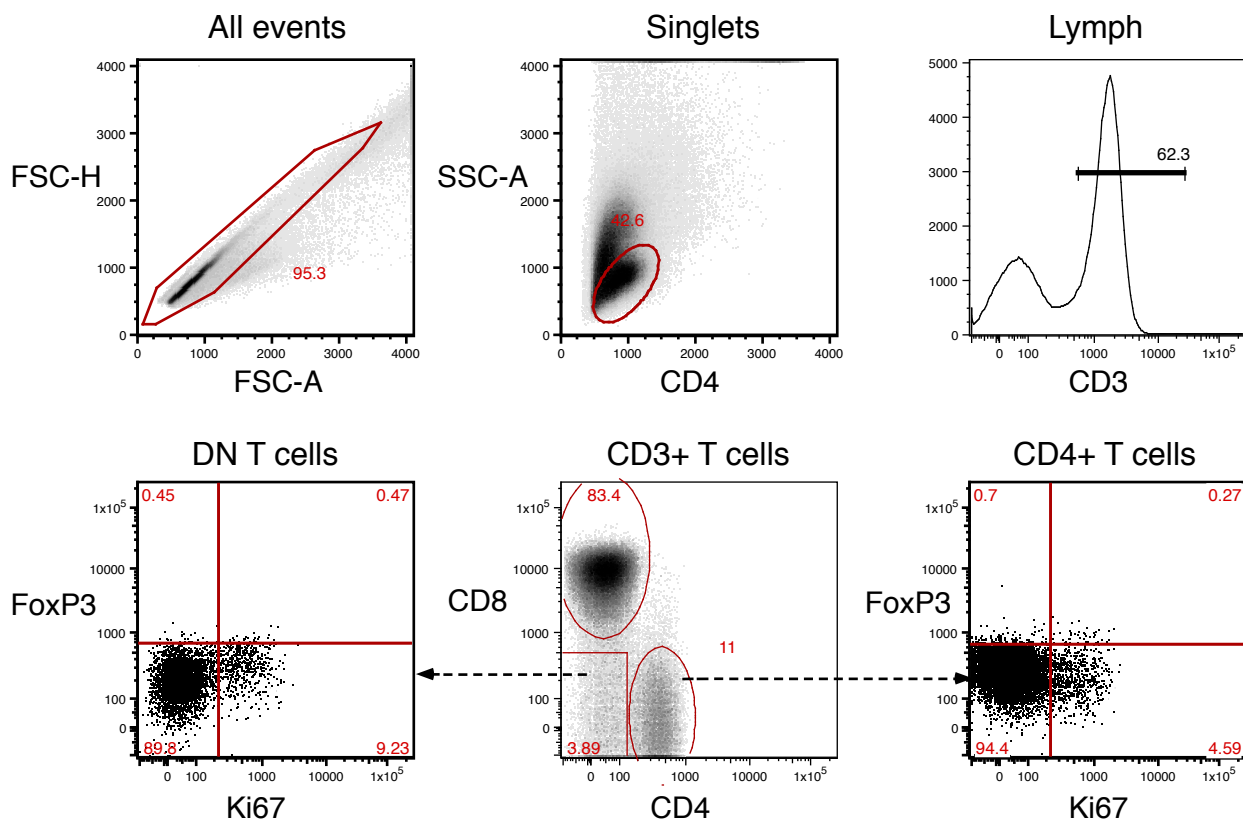

Ax. LN

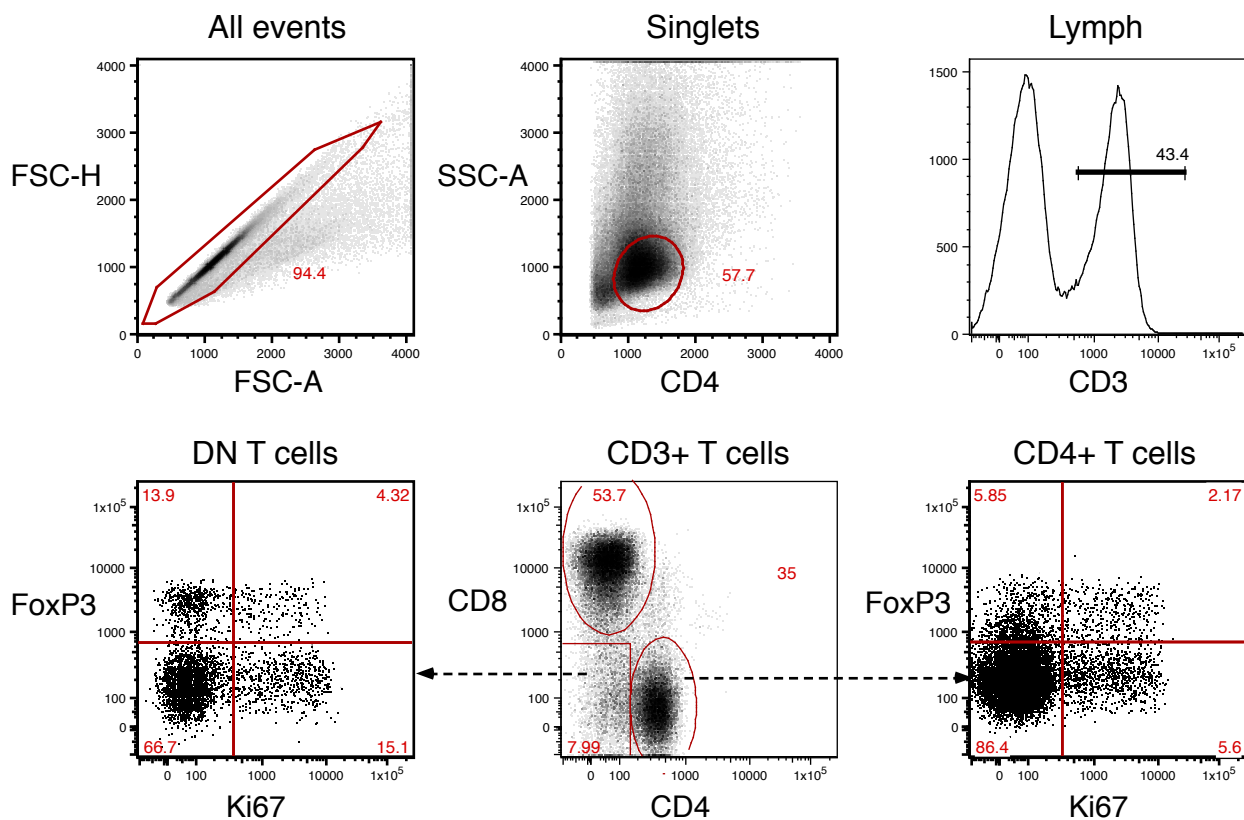

AGM

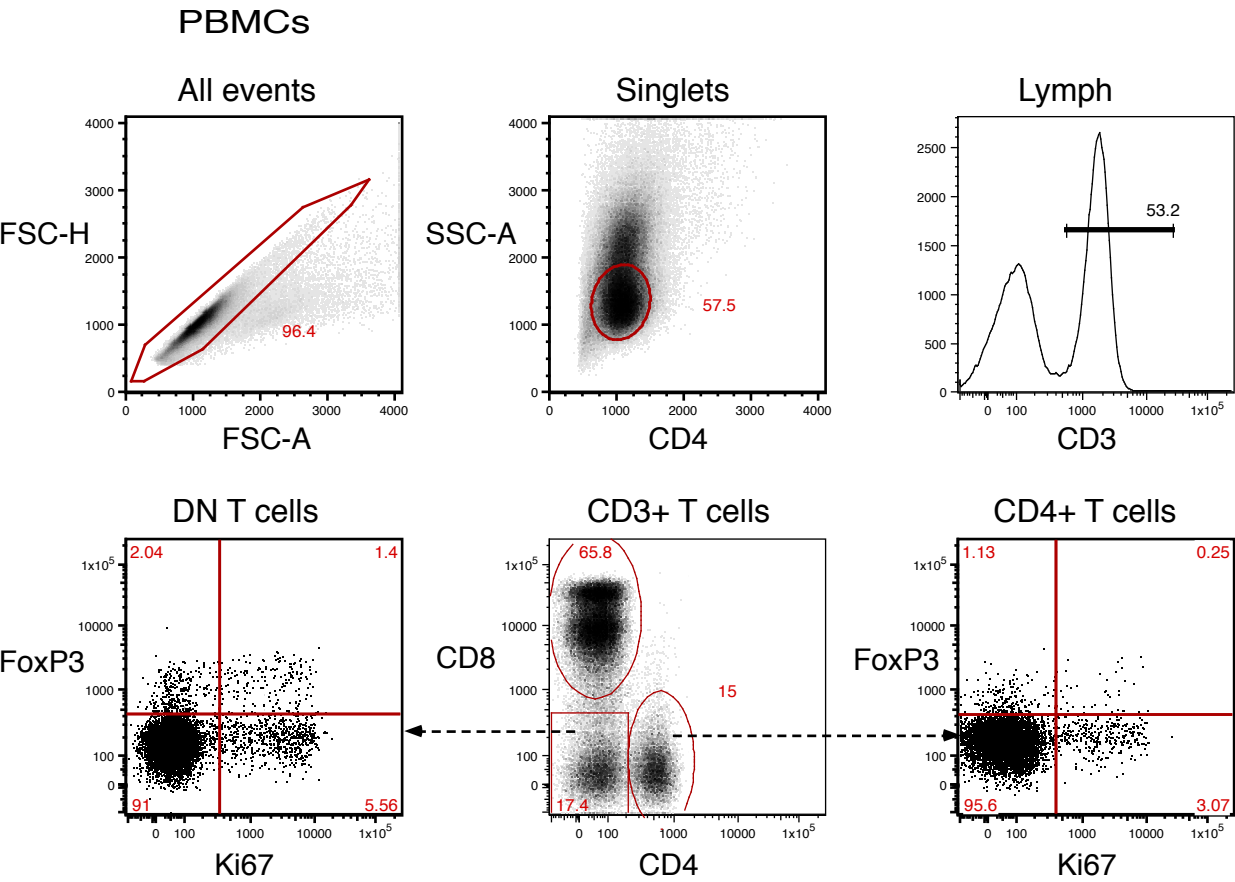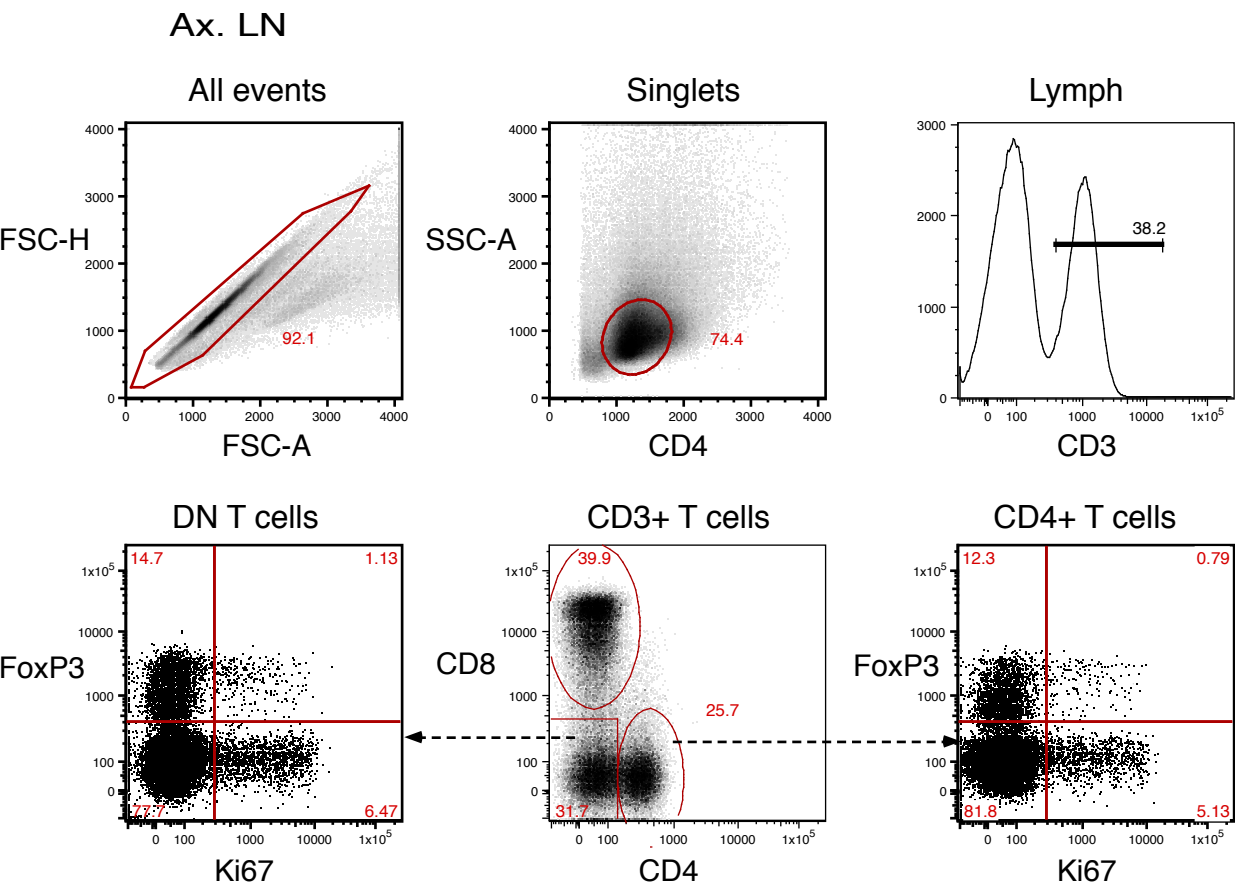

G

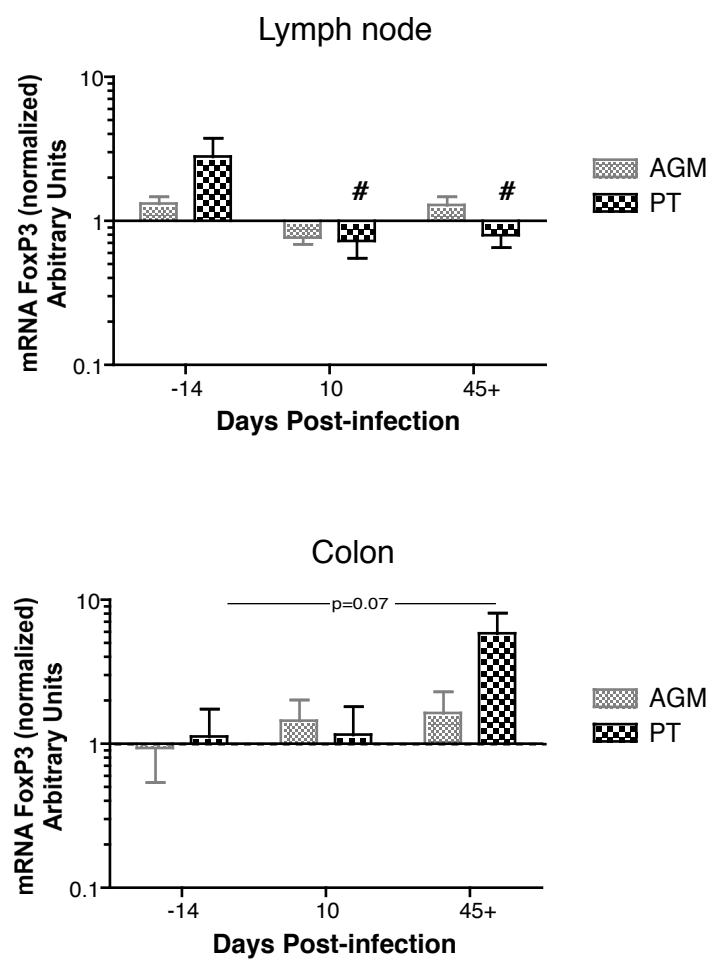

Supplement: Figure S3 — FoxP3+ T cell populations in the PT and AGM, pre- and post-infection. (A) FoxP3 and CD127 (IL-7 receptor) expression on CD4+ T cells in PBMCs [from a chronically SIVagm.sab92018-infected AGM (18 months post-infection)]. (B) CD25-enriched (“CD25+ Pos. Selection”) fractions using anti-CD25 magnetic beads, stimulated in vitro, and then analyzed for expression of IL-17, IL-2 or IFNγ, and FoxP3. (C–D) Suppression of cell division by total CD25+ Tregs from PBMCs [from chronically SIVagm.sab92018-infected AGMs (18 months post-infection)] on CFSE labeled CD25− PBMCs (Responder) gated on CD4+ T cells or CD8+ T cells with or without Treg add-back at a ratio of 1∶4 with responder cells (C-left). The same donor was tested with a dynamic range of Treg over responding cells with the frequency of undivided fraction indicated in CD4+ T cells or CD8+ T cells (C-right), showing augmented frequency of undivided cells as Treg/responder ratio increased. The same results were obtained when proliferation was analyzed using the FlowJo proliferation platform (D) from another AGM [% of Divided cells, Division index and Proliferation index], with data points indicating the % divided, proliferation index or division index achieved by CFSE-labeled cells in each sample as a percentage of the maximal value obtained in the CD25-depleted population with no add-back. (E. F) Simultaneous FoxP3 and Ki67 expression in CD4+ and DN T cells in the peripheral blood (upper) and axillary lymph node (lower) of a representative PT (A99059) (E) and AGM (06004) (at day 45+) (F). Also shown in each panel is the gating strategy for singlets, lymphocytes and CD3+ T cells. (G) FoxP3 mRNA levels in lymph node (upper) and colon (lower), as measured by real-time RT-PCR for FoxP3, with P values indicated in the PT lymph node and colon compared to baseline. (5.66 MB PDF) [file ppat.1000295.s003.pdf]
