# Supplementary material for: Critical Loss of the Balance between Th17 and T Regulatory Cell Populations in Pathogenic SIV Infection
Source: PLoS Pathog. 2009 Feb 13;5(2):e1000295. doi: 10.1371/journal.ppat.1000295 (PMC2635016; doi:10.1371/journal.ppat.1000295)
Supplement: Figure S2 — Changes in the frequency of multifunctional CD4+ and CD8+ T cells as a function of time post-infection in PTs and AGMs. (A) The frequency of multifunctional CD4+ T cells producing IL-2, TNFα, IFNγ, IL-17, and/or MIP1β was assessed by 10-color flow cytometry, analyzing cells secreting all 32 possible combinations of 1, 2, 3, 4 and/or 5 cytokines, with Boolean gating (FlowJo platform) and SPICE software (M. Roederer; described in Protocol S1). Rectangles R1, R2, and R3 highlight changes in the frequency of certain subpopulations that occurred over time primarily in the PT but not the AGM, whereas rectangle R4 highlights changes that occurred primarily in the AGM. Rectangles A, B, and C highlight IL-17+ subpopulations that also express IL-2 and TNFα (A), IL-2 alone (B), or no other cytokine from this list (C). Individual animals are represented by dots, means by dashed lines, and interquartile ranges by bars. P values (<0.05) were obtained using the Student's t-test (+) or Wilcoxon-Rank test (#), comparing mean values to the baseline values (day 0) for each species, as provided by SPICE software. (B) FACS detection of IL-17 (x-axis) and IL-2, TNFα, IFNγ, or MIP1β (y-axis) within CD3+CD4+ T cells over time in a representative PT (A99073) and AGM (06004) after PMA and ionomycin stimulation in vitro. (C) Differential representation of IL-17 (x-axis) and IL-2, TNFα, IFNγ, or MIP1β (y-axis) within CD3+CD4+ T cells in the peripheral blood, spleen and mesenteric lymph nodes of one representative PT (A99028) and one AGM (06005) at necropsy (day 45+). (1.31 MB PDF) [file ppat.1000295.s002.pdf]

Changes in Th17/Th1 multifunctional CD4+ T cell profiles during acute SIV infection (PBMCs)

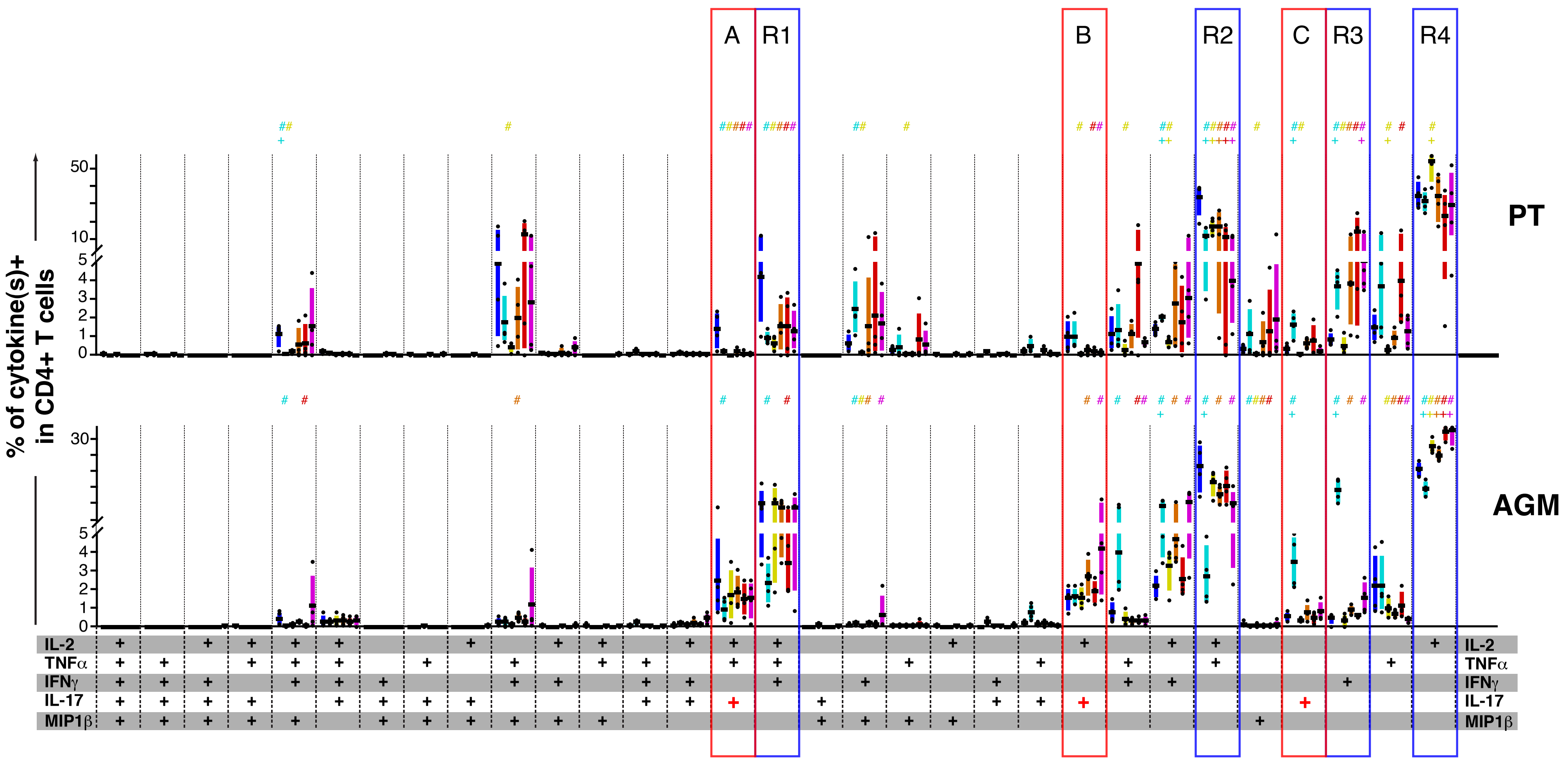

Timeline (days post SIVagm Infection)

0 3 10 17 28 45+

Statistical analysis (showed for cytokine(s) ≥1% of CD4+ T cells)

+: Student's t-test $p<0.05$ (Compared to Day0)

#: Wilcoxon-Rank $p<0.05$ (Compared to Day0)

Figure S2A

Figure S2

B

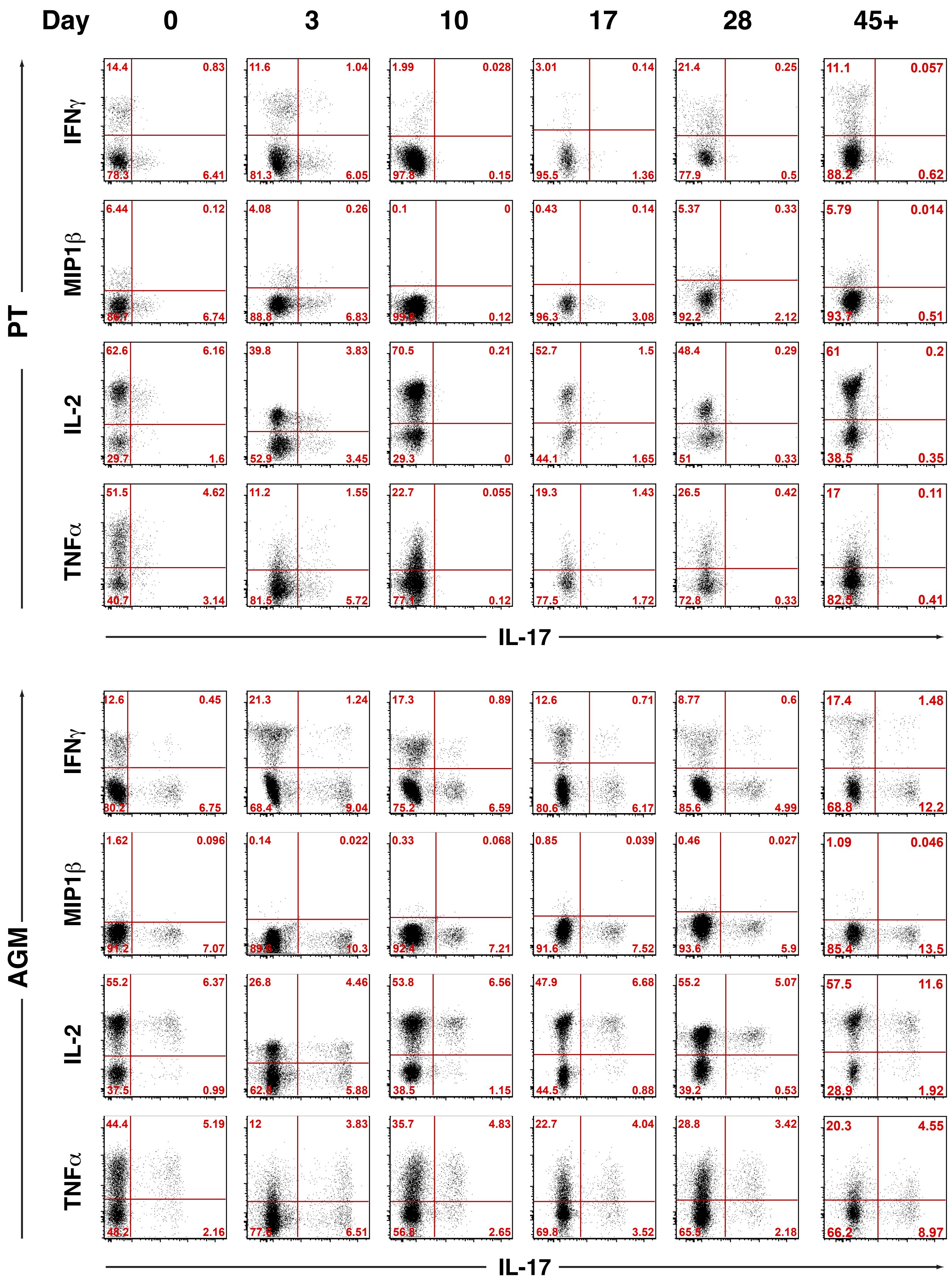
Day
0
3
10
17
28
45+
PT
AGM
IFNγ
MIP1β
IL-2
TNFα
IL-17
IL-17
14.4 0.83 78.3 6.41
11.6 1.04 81.3 6.05
1.99 0.028 97.8 0.15
3.01 0.14 95.5 1.36
21.4 0.25 77.9 0.5
11.1 0.057 88.2 0.62
6.44 0.12 86.7 6.74
4.08 0.26 88.8 6.83
0.1 0 99.8 0.12
0.43 0.14 96.3 3.08
5.37 0.33 92.2 2.12
5.79 0.014 93.7 0.51
62.6 6.16 29.7 1.6
39.8 3.83 52.9 3.45
70.5 0.21 29.3 0
52.7 1.5 44.1 1.65
48.4 0.29 51 0.33
61 0.2 38.5 0.35
51.5 4.62 40.7 3.14
11.2 1.55 81.5 5.72
22.7 0.055 77.1 0.12
19.3 1.43 77.5 1.72
26.5 0.42 72.8 0.33
17 0.11 82.5 0.41
12.6 0.45 80.2 6.75
21.3 1.24 68.4 9.04
17.3 0.89 75.2 6.59
12.6 0.71 80.6 6.17
8.77 0.6 85.6 4.99
17.4 1.48 68.8 12.2
1.62 0.096 91.2 7.07
0.14 0.022 89.6 10.3
0.33 0.068 92.4 7.21
0.85 0.039 91.6 7.52
0.46 0.027 93.6 5.9
1.09 0.046 85.4 13.5
55.2 6.37 37.5 0.99
26.8 4.46 62.6 5.88
53.8 6.56 38.5 1.15
47.9 6.68 44.5 0.88
55.2 5.07 39.2 0.53
57.5 11.6 28.9 1.92
44.4 5.19 48.2 2.16
12 3.83 77.8 6.51
35.7 4.83 56.8 2.65
22.7 4.04 69.8 3.52
28.8 3.42 65.9 2.18
20.3 4.55 66.2 8.97

Figure S2

C

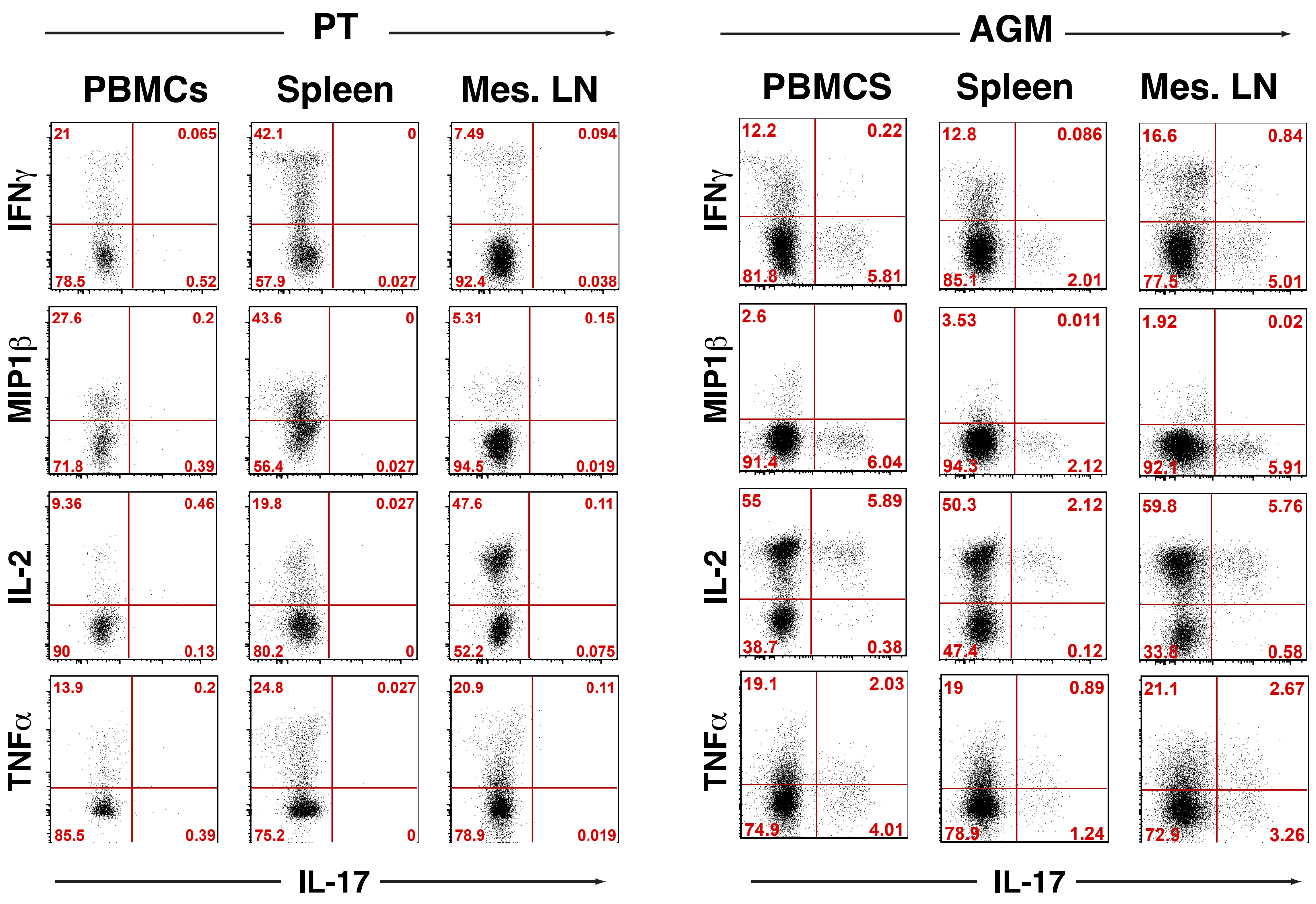

PT
AGM
PBMCs
Spleen
Mes. LN
PBMCS
Spleen
Mes. LN
IFNγ
MIP1β
IL-2
TNFα
IL-17
IL-17
