# Supplementary material for: Critical Loss of the Balance between Th17 and T Regulatory Cell Populations in Pathogenic SIV Infection
Source: PLoS Pathog. 2009 Feb 13;5(2):e1000295. doi: 10.1371/journal.ppat.1000295 (PMC2635016; doi:10.1371/journal.ppat.1000295)
Supplement: Protocol S1 — (0.10 MB DOC) [file ppat.1000295.s004.doc]

**PROTOCOL S1**

**Virus**

The primary isolate SIVagm.sab92018 (Dakar) was kindly provided by Dr. O. Diop at the Pasteur Institute in Dakar, Senegal [1]. This primary isolate has been used and described in a large number of studies [1,2,3,4,5,6,7,8,9,10]. Our viral stock was obtained by infecting one SIV negative C. sabaeus (Caribbean origin from the colony at the Center for Primate Neuroethology and Neuropsychiatric Institute, University of California, Los Angeles) with 300 50% tissue culture infectious doses (TCID_50_) of SIVagm.Sab92018 (Dakar), and by collecting plasma at day +10 following IV inoculation. The virus stock (pure plasma) was titrated on SupT1 cells at a titer of 1250 TCID_50_/ml, corresponding to 1.06x10^9^ RNA copies/ml (as measured by qRT-PCR; see below) and 7 ng/ml p27 (as measured by p27 ELISA, Zeptometrix, Buffalo, NY) as described in [1] and in Methods.

**SIVagm infection of non-human primates and tissue collection**

The AGMs had been transferred from the Wisconsin National Primate Research Center, Madison, WI, and had originated from St. Kitts. Aged-matched PTs were obtained from the WaNPRC colony. All 8 animals were quarantined and acclimated in the same facility at the WaNPRC. Multiple immunologic and gene expression parameters were analyzed at baseline, 14 days prior to infection (day -14). For colon biopsies, a Pentax® EC-340 endoscope was used to obtain 12-16 specimens from the rectal-sigmoid, descending, and transverse colon. Biopsies of inguinal lymph nodes were obtained under surgical procedure with complete anesthesia. Bone marrow aspirates [1 ml in a 3 ml syringe containing 0.25 ml citrate-based anticoagulant (ACD Solution A, BD Bioscience, San Jose, CA)] were obtained from the manubrium with an 18-21 G needle and controlled for quality (see next paragraph below). For these procedures, all animals were placed on prophylactic antibiotic therapy following biopsies at days -14 and 10 (Cefazolin IM 25 mg/kg followed by 10 consecutive days of Cephalexin 25 mg/kg BID). At necropsy, in addition to paired aspirates from bone marrow and biopsies from lymph nodes and colon, samples of tissue were also collected from iliac and mesenteric lymph nodes, tonsil, spleen, thymus, duodenum, jejunum, and ileum. For consistency and standardization, blood, bone marrow and tissue samples were collected and processed for analysis from all 8 animals on days -14 and 10. At necropsy, the cohort was divided into two groups, with half of the animals (2 PTs and 2 AGMs) processed on day 45 and the other half on day 49.

Whole blood was collected into EDTA-containing tubes (BD Biosciences, San Jose, CA) for cell counts, into ACD-containing tubes (BD Biosciences) for purification of PBMCs, or into PAXgene tubes (Qiagen, Valencia, CA) for RNA extraction. Sternal bone marrow (0.75 ml) was aspirated in a syringe containing ACD (0.25 ml) and kept on ice pending analysis. Lymph node and colon mucosal biopsies were immediately collected and distributed into either ice-cold tissue culture media (for subsequent cell extraction), PBS-buffered 4% formalin and snap-frozen in OCT (for histology), or RNAlater (Ambion, Austin, Tx) (for RNA extraction). PBMCs, LNCs, and colon cells were purified by using ficoll gradient (Histopaque, Sigma-Aldrich, St. Louis, MO) for blood, dispersion on filters (BD Biosciences) for lymph nodes and after collagenase treatment (Sigma-Aldrich) for gut cells.

Gut cells from colon biopsies or gut tissues (necropsy) were obtained after 3 successive 0.5 mg/ml collagenase type II treatments (Sigma-Aldrich, St Louis, MO), as previously described [11]. Cell viability in all samples was assessed by trypan blue exclusion. Blood contamination at the time of bone marrow aspiration was controlled by smearing a drop of bone marrow onto slides prior to aspiration and, later, ruled out by FACS analysis to show that all samples were comprised of <15% (5% on average) T cells.

**Expression microarray and real-time RT-PCR**

RNA concentrations from whole blood, PBMCs, and tissue samples were processed for microarray, as described [12,13,14]. Two selected sets of genes (“IFNα-induced genes” and “cytokine and chemokine genes”) were analyzed for fold-change over baseline at days 10 and 45+ in the two groups, including all 4 AGMs or 4 PTs.

The set of IFNα-induced genes included: IFNA1 (NM_024013); IFNA2 (NM_000605); IFNA4 (NM_021068); IFNA5 (NM_002169); IFNA6 (NM_021002); IFNA7, IFNA-J (NM_021057); IFNA8 (NM_002170); IFNA10 (NM_002171); IFNA13 (NM_006900); IFNA14 (NM_002172); IFNA16 (NM_002173); IFNA17 (NM_021268); IFNB1 (NM_002176); IFNG (NM_000619); IFNB1 (NM_002176); IFNW1 (NM_002177); IFNAR1 (NM_000629); IFNAR2 (NM_000874); IFNGR1 (NM_000416); IFNGR2 (NM_005534); OAS1 (NM_002534; NM_016816); OAS2 (NM_002535; NM_016817); OAS3 (NM_006187); HLA-A, B, C (NM_002116; NM_005514; NM_002117); IRF1, 2,3,5,7 (NM_002198; NM_002199; NM_001571; NM_002200; NM_001572); ISG20 (NM_002201); ISGF3G (NM_006084); INDO (NM_002164); GADD45A (NM_001924); MX1 (NM_002462); MX2 (NM_002463); PML (NM_033247); ADAR (NM_001111); IFI16 (NM_005531); IFI27 (NM_005532); IFI30 (NM_006332); IFI35 (NM_005533); IFI44 (NM_006417); IFI44L (NM_006820); IFIH1 (NM_022168); IFIT1, 2, 3,5 (NM_001548; NM_001547; NM_001549; NM_012420); IFITM1 (NM_003641); IFITM2 (NM_006435); DDX58 (RIG-I) DEAD (Asp-Glu-Ala-Asp) box polypeptide 58 (NM_01431; NM_014314). In this set of genes, only those genes showing greater than 99% probability of being differentially expressed (p ≤ 0.01) and an expression level change of 2-fold or greater in at least one of the error-weighted combines [AGM group (n=4) or PT group (n=4)] for colon or blood on either day 10 or day 45+ were included for transcriptional profiling, as presented in Figure 2B and Table S1A.

The set of cytokine and chemokine genes included: IL-1A (NM_000575); IL-1B (NM_000576); IL-2 (NM_000586); IL-3 (NM_000588); IL-4 (NM_172348); IL-5 (NM_000879); IL6 (NM_000600); IL-7 (NM_000880); IL-8 (NM_000584); IL-9 (NM_000590); IL-10 (NM_000572); IL-12A (NM_000882); IL-12B (NM_002187); IL-13 (NM_002188); IL-15 (NM_172174); IL-16 (NM_004513; NM_172217); IL-17A (NM_002190); IL-17E, IL-25 (NM_022789); IL-17F (NM_052872); IL-18 (NM_001562); IL-21 (NM_021803); IL-22 (NM_020525); IL-23A, P19 (NM_016584); IL-24 (NM_006850); IL-27 (NM_145659); TGFB1I1 (NM_015927); TGFB1 (NM_000660); TGFB2 (NM_003238); TGFB3 (NM_003239); CCL2 (MCP-1) (NM_002982); CCL3, MIP-1-alpha (NM_002983); CCL4, MIP-1-beta (NM_002984); CCL5, RANTES (NM_002985); CCL17 (TARC) (NM_002987); CCL18 (NM_002988); CCL19 (NM_006274); CCL20 (NM_004591); CCL21 (NM_002989); CCL22 (NM_002990); CCL23 (NM_005064); CCL24 (NM_002991); CCL25 (NM_148888; NM_005624); CCL26 (NM_006072); CCL27 (NM_006664); CCL28 (NM_019846); CXCL1 (NM_001511); CXCL2, MIP2A (CINC) (NM_002089); CXCL3, MIP2B (NM_002090); CXCL5 (NM_002994); CXCL6 (NM_002993); CXCL9 (MIG) (NM_002416); CXCL10 (IP-10) (NM_001565); CXCL11 (IP-9) (NM_005409); CXCL12, SDF-1 (NM_000609; NM_199168); CXCL13 (NM_006419); CXCL14 (NM_004887); CXCL16 (NM_022059); CX3CL1 (fractalkine) (NM_002996); XCL1,LTN (NM_002995); XCL2 (NM_003175); XCR1 (NM_005283); LIF (NM_002309); S100A8 (NM_002964); S100A9 (NM_002965); MCSF (NM_172211; NM_172212); GMCSF (NM_000758); TNF (NM_000594); PTGS1 (NM_000962); PGFS2 (NM_000963). In this set of genes, only those showing greater than 99% probability of being differentially expressed (p ≤ 0.01) and an expression level change of 2-fold or greater in at least one of the error-weighted combines [AGM group (n=4) or PT group (n=4)] for colon on either day 10 or day 45+ were included for transcriptional profile as presented in Figure 2C and Table S1B).

For validation of gene expression by RT-PCR, all primer-probe sets were controlled for efficiency by serial dilution of RT products from control RNA extracts from lymph nodes of both PTs and AGMs. Similarly, each experimental run included a control for efficiency using an HPRT primer-probe set and serial dilution of RT control sample. Experiments were considered conclusive only when the log transformed efficiency curve from control samples was linear and the slope was -3.3 +/-10%. All sequences were amplified using the 7900 default amplification program: 2 min at 50°C, 10 min at 95°C, followed by 50 cycles of 15 s at 95°C and 1 min at 60°C. The results were analyzed with the SDS 7900 system software, version 2.1 (Applied Biosystems).

**Cell counts, phenotype, and functional analysis by flow cytometry**

All antibodies were titrated and characterized on AGM and rhesus macaque cells prior to use. All staining procedures, with the exception of those carried out in Trucount tubes, were performed in 96-well V bottom plates. Cells were subsequently transferred into 96-well U-bottom plates for FACS analysis using a High Throughput System (HTS) (Becton Dickinson, San Jose, CA).

After complete blood counts (CBC) were determined at the WaNPRC Clinical Lab in Seattle, counts of B, CD4^+^, CD8^+^, or CD3^+^CD4^-^CD8^-^ double-negative (DN) cell populations as well as of their memory/effector/naïve subpopulations were determined independently using two different methods: by multiplying the lymphocyte counts (from the CBC) times the frequency of any given population in the lymphocyte gate (as determined by FACS analysis with Panel 1, see Table S2); and by absolute counts obtained on 50 µl whole blood using Trucount absolute counting tubes (Becton Dickinson, San Jose, CA) and Panel 4. Both methods gave identical numbers within a 10% margin of error. Cells counts in bone marrow were determined on Trucount tubes only, using 10 µl of bone marrow suspension (Panel 5).

Following purification and/or extraction, PBMCs, mononuclear lymph node and colon cells as well as all tissue cells obtained at necropsy, were counted and resuspended at 5x10^6^ cells/ml in R-10 media for subsequent *in vitro* stimulation or phenotyping.

Phenotyping was performed by cell surface staining of 3-5x10^5^ cells in FACS buffer (PBS, 2% FBS, 0.5 mM EDTA) and, as specified, by intracellular staining (Fix/Perm and Perm/wash buffers, BD or FoxP3 buffer set, Biolegend, San Diego, CA). Panels 1, 3, and 8 (the later at necropsy only) were used on PBMCs and mononuclear cells from lymph node and colon biopsies as well as necropsy tissues to characterize T cell subpopulations, using antibodies specific for differentiation markers (CD45RA and CD27), activation markers (Ki67, HLA-DR, MHC-I, CCR5, CD69, CD25, PD-1), chemokine receptors (CCR5 and CCR9), apoptotic cells (AnnexinV), and Tregs (CD25^+^, FoxP3^+^). Populations from peripheral blood and lymph node were divided into subpopulations of naïve (CD45RA^+^CD27^+^), memory (CD45RA^-^CD27^+^), terminally differentiated effector (CD45RA^+^CD27^-^), and effector (CD45RA^-^CD27^-^) T cells. Panels 6 and 7 were used on whole cell populations after non-fixative red blood cell lysis (ACK lysing buffer, Invitrogen). All steps using Panels 2 and 7 (AnnexinV staining) were carried out in Ca^2+^ and Mg^2+^ rich FACS buffer (PBS, 2.5 mM CaCl_2_ and MgCl_2_, FBS 2%) and cells were fixed in 1% PFA at the end in the same buffer. Multifunctional cytokine assays were performed using panels 9 and 10 (see Table S2) on PBMCs and LN cells, and Panel 11 on colon cells and other necropsy samples at all time points with the exception of day -14, for which a prior Panel 11 (lacking anti-IL-17 antibodies) was used instead. We determined the frequency of Th17 cells in blood and tissues by intracellular cytokine detection of the Th17-defining cytokine IL-17A in T cells stimulated with PMA and ionomycin. All staining for intracellular phenotyping (Panel 1, 3, and 6) and cytokine (Panel 9, 10 and 11) were performed in a two-step manner, e.g. surface and then intracellular staining, using Cytofix/perm and Perm/wash buffer set (BD Bioscience, Panels 1, 6, 9, 10, 11) or a FoxP3 buffer (Biolegend, San Diego, CA, Panel 3), as previously described [15] or according to the manufacturers’ instructions.

Due to background FoxP3 staining in some animals/time points, the FoxP3 gating strategy used in Figure 5 and Figure S3 was based on: (1) the detection of a distinct positive FoxP3^+^ population in CD4+ T cells (or memory/effector CD4^+^ T cells), (2) the background level of FoxP3^+^ expression in other subset CD8+ T cells and CD3^-^CD8^-^ lymphocytes, and (3) the comparison of (1) and (2) in other tissues from the same animal and time point when available (e.g., lymph nodes at day -14, day 10 and day45+, and other tissues at necropsy). This gating strategy is detailed in Figures S3E and S3F. In most cases, the same gating of FoxP3^+^ events among CD4^+^ T lymphocytes was reported in all animals from the same time point. However, in rare occasions (see, for example, PT99059 at day 3 and day 45^+^ in Figure 5C), higher background of FoxP3 staining was noticed in CD4+ T cells and also in other classically non-Treg cell populations (CD8^+^ T cells, CD3- negative lymphocytes, data not shown). In addition, a distinct FoxP3^+^ population above background was clearly identified from the same animal (PT99059, Day 45^+^), when lymph node cells (axillary, inguinal and mesenteric lymph nodes) were stained at the same time [(Figure S3E: compare PBMCs (upper) and lymph node cells (lower)]. In such cases, the threshold for FoxP3-positive events in CD4^+^ T cells from PBMCs (Figure 5C) was higher than the other animals.

*In vitro* Treg suppression assays were performed as described[16,17], after polyclonal stimulation using anti-CD3 coated 96-well plates (clone SP34-2 coated with 50 µl of 2 µg/ml [anti-CD3]) and soluble anti-CD28 (clone 28.2, 0.5 µg/ml). Responder cells were prepared by CD25-negative depletion of purified PBMCs using anti-CD25–labeled paramagnetic microbeads and LS columns, according to the manufacturer's instructions (Miltenyi Biotec). Cells were labeled with CFSE (1 µM). Candidate Treg cells were prepared by positive selection on MS columns. The purity and cell composition were assessed by using the phenotypic Treg panel (panel 3, Supplementary Table 2, with CD127 instead of PD1), showing >90% CD25^+^FoxP3^+^ depletion or significant enrichment of CD25^+^FoxP3^+^ T cells after negative or positive selection respectively (data not shown). A minimum of 200,000 responder cells was added to antibody-coated wells of a 96-well plate, alone or together with a variable number of candidate regulatory cells. Plates were placed in the incubator for 5 d before harvesting, staining, and analysis using the LSRII (BD Biosciences) and FlowJo software. For analysis of CD4^-^CD8^-^ double-negative Tregs, the same protocol was used after positive selection on CD25^+^ cells but on cells that were first depleted of CD4^+^ T cells by anti-CD4–labeled paramagnetic microbeads and LS columns.

*In vitro* suppression assays were analyzed on FlowJo software v6-8 (Treestar, Ashland, OR), proliferation platform. The parameters analyzed are described on the FlowJo website (see <http://www.flowjo.com/v8/html/proliferation.html>): “%Divided” is the percentage of the cells of the original sample which divided (assuming that no cells died during the culture); for example, if half of the cells in the starting population have divided, the %Divided = 50%. “Proliferation Index” is the average number of divisions that those cells which divided underwent. For example, if the average number of divisions for all the cells was 4, the Division Index would be 4. “Division Index” is the average number of divisions that a cell (that was present in the starting population) has undergone. These statistics are related in the following way: Division Index = (Prolif. Index)(%Divided).

Multifunctional cytokine analysis was performed using FlowJo by stringent gating of each cytokine positive population after PMA/Iono stimulation (with corresponding unstimulated samples <0.01% positive events) and subsequent Boolean gating (FlowJo), as described [18]. Boolean values representing the frequency of cells secreting 1, 2, 3, and/or 4 cytokines in each cell population (e.g., CD4^+^ or CD8^+^ T cells, CD3^+^CD4^-^CD8^-^ DN cells, and naïve, memory and effector subpopulations among singlets/live/CD3^+^ lymphocytes) were reported in SPICE as background-subtracted values compared to unstimulated samples for each cell population of each sample considered. Threshold values were determined for the combination of 1-4 cytokines in CD4^+^, CD8^+^, and DN cells in PBMCs, or LN cells as the absolute value of the 95^th^ percentile of the distribution of negative value after summing (when 4 or less cytokines were analyzed) on a per cell population, per AGM or PT macaque group, per tissue (PBMCs or LNCs) and per stimulus for all time points.

**REFERENCES RELATED TO PROTOCOL S1**

1. Diop OM, Gueye A, Dias-Tavares M, Kornfeld C, Faye A, et al. (2000) High levels of viral replication during primary simian immunodeficiency virus SIVagm infection are rapidly and strongly controlled in African green monkeys. J Virol 74: 7538-7547.

2. Gueye A, Diop OM, Ploquin MJ, Kornfeld C, Faye A, et al. (2004) Viral load in tissues during the early and chronic phase of non-pathogenic SIVagm infection. J Med Primatol 33: 83-97.

3. Ploquin MJ, Diop OM, Sol-Foulon N, Mortara L, Faye A, et al. (2004) DC-SIGN from African green monkeys is expressed in lymph nodes and mediates infection in trans of simian immunodeficiency virus SIVagm. J Virol 78: 798-810.

4. Kornfeld C, Ploquin MJ, Pandrea I, Faye A, Onanga R, et al. (2005) Antiinflammatory profiles during primary SIV infection in African green monkeys are associated with protection against AIDS. J Clin Invest 115: 1082-1091.

5. Pandrea I, Kornfeld C, Ploquin MJ, Apetrei C, Faye A, et al. (2005) Impact of viral factors on very early in vivo replication profiles in simian immunodeficiency virus SIVagm-infected African green monkeys. J Virol 79: 6249-6259.

6. Pandrea I, Apetrei C, Dufour J, Dillon N, Barbercheck J, et al. (2006) Simian immunodeficiency virus SIVagm.sab infection of Caribbean African green monkeys: a new model for the study of SIV pathogenesis in natural hosts. J Virol 80: 4858-4867.

7. Ploquin MJ, Desoutter JF, Santos PR, Pandrea I, Diop OM, et al. (2006) Distinct expression profiles of TGF-beta1 signaling mediators in pathogenic SIVmac and non-pathogenic SIVagm infections. Retrovirology 3: 37.

8. Pandrea IV, Gautam R, Ribeiro RM, Brenchley JM, Butler IF, et al. (2007) Acute loss of intestinal CD4+ T cells is not predictive of simian immunodeficiency virus virulence. J Immunol 179: 3035-3046.

9. Diop OM, Ploquin MJ, Mortara L, Faye A, Jacquelin B, et al. (2008) Plasmacytoid dendritic cell dynamics and alpha interferon production during Simian immunodeficiency virus infection with a nonpathogenic outcome. J Virol 82: 5145-5152.

10. Pandrea I, Gaufin T, Brenchley JM, Gautam R, Monjure C, et al. (2008) Cutting edge: Experimentally induced immune activation in natural hosts of simian immunodeficiency virus induces significant increases in viral replication and CD4+ T cell depletion. J Immunol 181: 6687-6691.

11. Shacklett BL, Yang O, Hausner MA, Elliott J, Hultin L, et al. (2003) Optimization of methods to assess human mucosal T-cell responses to HIV infection. J Immunol Methods 279: 17-31.

12. Kash JC, Muhlberger E, Carter V, Grosch M, Perwitasari O, et al. (2006) Global suppression of the host antiviral response by Ebola- and Marburgviruses: increased antagonism of the type I interferon response is associated with enhanced virulence. J Virol 80: 3009-3020.

13. Kash JC, Tumpey TM, Proll SC, Carter V, Perwitasari O, et al. (2006) Genomic analysis of increased host immune and cell death responses induced by 1918 influenza virus. Nature 443: 578-581.

14. Kobasa D, Jones SM, Shinya K, Kash JC, Copps J, et al. (2007) Aberrant innate immune response in lethal infection of macaques with the 1918 influenza virus. Nature 445: 319-323.

15. Emu B, Sinclair E, Favre D, Moretto WJ, Hsue P, et al. (2005) Phenotypic, functional, and kinetic parameters associated with apparent T-cell control of human immunodeficiency virus replication in individuals with and without antiretroviral treatment. J Virol 79: 14169-14178.

16. Aandahl EM, Michaelsson J, Moretto WJ, Hecht FM, Nixon DF (2004) Human CD4+ CD25+ regulatory T cells control T-cell responses to human immunodeficiency virus and cytomegalovirus antigens. J Virol 78: 2454-2459.

17. Hartigan-O'Connor DJ, Abel K, McCune JM (2007) Suppression of SIV-specific CD4+ T cells by infant but not adult macaque regulatory T cells: implications for SIV disease progression. J Exp Med 204: 2679-2692.

18. Precopio ML, Betts MR, Parrino J, Price DA, Gostick E, et al. (2007) Immunization with vaccinia virus induces polyfunctional and phenotypically distinctive CD8(+) T cell responses. J Exp Med 204: 1405-1416.
