# Supplementary material for: Critical Loss of the Balance between Th17 and T Regulatory Cell Populations in Pathogenic SIV Infection
Source: PLoS Pathog. 2009 Feb 13;5(2):e1000295. doi: 10.1371/journal.ppat.1000295 (PMC2635016; doi:10.1371/journal.ppat.1000295)
Supplement: Table S1 — IFNα-induced and cytokine/chemokine transcripts analyzed in the arrays of Figure 2, B and C (see Protocol S1 for more details). (0.21 MB PDF) [file ppat.1000295.s005.pdf]

Table S1

A. Interferon-α (IFNα)-induced Genes

Primary Sequence Name	Accession #	Sequence Description
IFNGR1	NM_000416	Interferon gamma receptor 1
IFNAR2	NM_207585	Interferon (alpha, beta and omega) receptor 2
IRF1	NM_002198	Interferon regulatory factor 1
ISGF3G	NM_006084	Interferon-stimulated transcription factor 3, gamma 48kDa
OAS2	NM_002535	2'-5'-oligoadenylate synthetase 2, 69/71kDa
OAS2	NM_016817	2'-5'-oligoadenylate synthetase 2, 69/71kDa
IRF7	NM_001572	Interferon regulatory factor 7
ISG20	NM_002201	Interferon stimulated exonuclease gene 20kDa
IFI35	NM_005533	Interferon-induced protein 35
IFITM2	NM_006435	Interferon induced transmembrane protein 2 (1-8D)
ADAR	NM_001111	Adenosine deaminase, RNA-specific
IFIT5	NM_012420	Interferon-induced protein with tetratricopeptide repeats 5
INDO	NM_002164	Indoleamine-pyrrole 2,3 dioxygenase
IFI16	NM_005531	Interferon, gamma-inducible protein 16
PML	NM_033247	Promyelocytic leukemia
OAS3	NM_006187	2'-5'-oligoadenylate synthetase 3, 100kDa
IFIT3	NM_001549	Interferon-induced protein with tetratricopeptide repeats 3
MX2	NM_002463	Myxovirus (influenza virus) resistance 2 (mouse)
MX1	NM_002462	Myxovirus (influenza virus) resistance 1, interferon-inducible protein p78 (mouse)
OAS1	NM_016816	2',5'-oligoadenylate synthetase 1, 40/46kDa
IFIH1	NM_022168	Interferon induced with helicase C domain 1
IFIT2	NM_001547	Interferon-induced protein with tetratricopeptide repeats 2
IFIT1	NM_001001887	Interferon-induced protein with tetratricopeptide repeats 1 (IFIT1)
IFI27	NM_005532	Interferon, alpha-inducible protein 27
IFI44	NM_006417	Interferon-induced protein 44

B. Cytokines/Chemokines

Primary Sequence Name	Accession #	Sequence Description
CCL28	NM_019846	Chemokine (C-C motif) ligand 28
CCL20	NM_004591	Chemokine (C-C motif) ligand 20
CXCL1	NM_001511	Chemokine (C-X-C motif) ligand 1 (melanoma growth stimulating activity, alpha)
CXCL2	NM_002089	Chemokine (C-X-C motif) ligand 2
IL1B	NM_000576	Interleukin 1, beta
IL22	NM_020525	Interleukin 22
S100A8	NM_002964	S100 calcium binding protein A8
S100A9	NM_002965	S100 calcium binding protein A9
CXCL9	NM_002416	Chemokine (C-X-C motif) ligand 9
CXCL10	NM_001565	Chemokine (C-X-C motif) ligand 10
CXCL11	NM_005409	Chemokine (C-X-C motif) ligand 11
CCL23	NM_005064	Chemokine (C-C motif) ligand 23
CCL2	NM_002982	Chemokine (C-C motif) ligand 2
CCL19	NM_006274	Chemokine (C-C motif) ligand 19
CCL21	NM_002989	Chemokine (C-C motif) ligand 21
CXCL6	NM_002993	Chemokine (C-X-C motif) ligand 6 (granulocyte chemotactic protein 2)
CXCL12	NM_199168	Chemokine (C-X-C motif) ligand 12 (stromal cell-derived factor 1)
CXCL12	NM_000609	Chemokine (C-X-C motif) ligand 12 (stromal cell-derived factor 1)
TGFB1I1	NM_015927	Transforming growth factor beta 1 induced transcript 1
TGFB2	NM_003238	Transforming growth factor, beta 2
TGFB3	NM_003239	Transforming growth factor, beta 3
PTGS1	NM_000962	Prostaglandin-endoperoxide synthase 1 (prostaglandin G/H synthase and cyclooxygenase)
PTGS2	NM_000963	Prostaglandin-endoperoxide synthase 2 (prostaglandin G/H synthase and cyclooxygenase)
CXCL14	NM_004887	Chemokine (C-X-C motif) ligand 14
CXCL13	NM_006419	Chemokine (C-X-C motif) ligand 13 (B-cell chemoattractant)

Table S1. IFNα-induced and cytokine/chemokine transcripts analyzed in the arrays of Figure 2, B and C
