# Supplementary material for: Critical Loss of the Balance between Th17 and T Regulatory Cell Populations in Pathogenic SIV Infection
Source: PLoS Pathog. 2009 Feb 13;5(2):e1000295. doi: 10.1371/journal.ppat.1000295 (PMC2635016; doi:10.1371/journal.ppat.1000295)
Supplement: Table S2 — Antibody panels for multiparameter flow cytometry. BD: BD Bioscience, San Jose, CA; Coulter: Beckman Coulter, Inc. Fullerton, CA, eBiosciences: eBiosciences, San Diego, CA; Biolegend: Biolegend, San Diego, CA, R&D: R&D Systems, Inc, Minneapolis, MN; Cedarlane: Cedarlane Laboratories Ltd. Burlington, NC; NPRR: National Institutes of Health (NIH) Nonhuman Primate Reagent Resource (NPRR) from K. Reimann (Harvard University, Cambridge, MA). CD38-FITC (OKT-10) is kind gift from R. Reyes, UCSF, San Francisco, CA. (0.34 MB PDF) [file ppat.1000295.s006.pdf]

Table S2

	Panel 1 Activation/Subpopulation				Panel 2 Apoptosis				Panel 3 Regulatory T cells/Activation			
Fluorescent dye	*Antigen*	*Clone*	*Manufact.*	Dilution	*Antigen*	*Clone*	*Manufact.*	Dilution	*Antigen*	*Clone*	*Manufact.*	Dilution
Pacific Blue	**CD3**	SP34-2	BD	100	**Live/Dead**		Invitrogen	3000	**CD3**	SP34-2	BD	100
FITC/Alexa488	**Ki67**	B56	BD	25	**Annexin V**		BD	100	**Ki67**	B56	BD	25
PE	**CD8**β	2ST8-5H7	Coulter	50	**MHC-I**	W6/32	Dako	100	**PD-1**	MIH4	BD	25
ECD	**CD45RA**	2H4	Coulter	50	**CD45RA**	2H4	Coulter	50	**CD45RA**	2H4	Coulter	50
Amcyan	**CD4**	L200	NPRR	25	**CD4**	L200	NPRR	25	**CD4**	L200	NPRR	25
APC/Alexa647	**CCR9**	112509	BD	25	**CCR5**	3A9	BD	25	**FoxP3**	206D	Biolegend	20
PE-Cy5.5	**CD8**	3b5	Invitroegn	200	**CD8**	3b5	Invitroegn	200	**CD8**	3b5	Invitroegn	200
PE-Cy7	**HLA-DR**	L243	BD	100	**CD20**	B9E9	BD	50	**CD25**	M-A251	BD	50
APC-Cy7/-Alexa750	**CD27**	O323	eBioscience	100	**CD27**	O323	eBioscience	100	**CD27**	O323	eBioscience	100
Alexa700					**CD3**	SP34.2	BD	100				

	Panel 4 Trucount Whole Blood (50μl)				Panel 5 Trucount Bone Marrow (10μl)				Panel 6 Activation, Bone marrow			
Fluorescent dye	*Antigen*	*Clone*	*Manufact.*	Dilution	*Antigen*	*Clone*	*Manufact.*	Dilution	*Antigen*	*Clone*	*Manufact.*	Dilution
Pacific Blue	**CD3**	SP34-2	BD	100	**Live/Dead**		Invitrogen	3000	**CD3**	SP34-2	BD	100
FITC/Alexa488	**CD38**	OKT10	(R.Reyes)	400	**CD38**	OKT10	(R.Reyes)	400	**Ki67**	B56	BD	25
PE	**CCR5**	3A9	Coulter	25	**CD34**	563	BD	25	**CD34**	563	BD	25
ECD	**CD45RA**	2H4	Coulter	50	**CD20**	B9E9	Coulter	50	**CD45RA**	2H4	Coulter	50
Amcyan					**CD4**	L200	NPRR	25	**CD4**	L200	NPRR	25
APC/Alexa647	**CD4**	L200	BD	25	**CCR5**	3A9	BD	25	**CCR5**	3A9	BD	25
PE-Cy5.5	**CD8**	3b5	Invitroegn	200	**CD8**	3b5	Invitroegn	200	**CD8**	3b5	Invitroegn	200
PE-Cy7	**CD20**	B9E9	BD	50	**CD25**	M-A251	BD	50	**HLA-DR**	L243	BD	100
APC-Cy7/-Alexa750	**CD27**	O323	eBioscience	100	**CD3**	SP34-2	BD	100	**CD27**	O323	eBioscience	100
Alexa700												

	Panel 7 Apoptosis, Bone marrow				Panel 8 Subpopulation-2			
Fluorescent dye	*Antigen*	*Clone*	*Manufact.*	Dilution	*Antigen*	*Clone*	*Manufact.*	Dilution
Pacific Blue	**Live/Dead**		Invitrogen	3000	**CD3**	SP34-2	BD	100
FITC/Alexa488	**Annexin V**		BD	100	**CD11a**	25.3	Coulter	25
PE	**CD34**	563	BD	25	**CCR7**	150503	R&D	100
ECD	**CD20**	B9E9	Coulter	50	**CD45RA**	2H4	Coulter	50
Amcyan					**CD4**	L200	NPRR	25
APC/Alexa647	**CD4**	L200	BD	25	**CD28**	CD28.2	BD	25
PE-Cy5.5	**CD8**	3b5	Invitroegn	200	**CD8**	3b5	Invitrogen	200
PE-Cy7	**CD25**	M-A251	BD	50	**CD69**	FN50	BD	100
APC-Cy7/-Alexa750	**CD69**	FN50	BD	50	**CD27**	3B5	eBioscience	100
Alexa700	**CD3**	SP34.2	BD	100				

	Panel 9 Th1 and Th17				Panel 10 Th2 and Treg				Panel 11 Th1, Th2 and Th17			
Fluorescent dye	*Antigen*	*Clone*	*Manufact.*	Dilution	*Antigen*	*Clone*	*Manufact.*	Dilution	*Antigen*	*Clone*	*Manufact.*	Dilution
Pacific Blue	**CD3**	SP34-2	BD	100	**Live/Dead**		Invitrogen	3000	**CD3**	SP34-2	BD	100
FITC/Alexa488	**MIP1**β	25.3	R&D	50	**IL-4**	MP4-25D2	Invitrogen	50	**IL-2**	MQ1-17H12	BD	50
PE	**IL-17**	eBio64CAP17	eBioscience	50	**TGF**β	TB21	Cedarlane	100	**TGF**β	TB21	Cedarlane	100
ECD	**CD45RA**	2H4	Coulter	50	**CD20**	2H4	Coulter	50	**CD45RA**	2H4	Coulter	50
Amcyan	**CD4**	L200	NPRR	50	**CD4**	L200	NPRR	25	**CD4**	L200	NPRR	25
APC/Alexa647	**IL-2**	MQ1-17H12	BD	50	**IL-10**	B-T10	Miltenyi	50	**IL-17**	eBio64CAP17	eBioscience	50
PE-Cy5.5	**CD8**	3b5	Invitrogen	200	**CD8**	3b5	Invitroegn	200	**CD8**	3b5	Invitrogen	200
PE-Cy7	**IFN**γ	B27	BD	50	**CD25**	M-A251	BD	50	**IFN**γ	B27	BD	50
APC-Cy7/-Alexa750	**CD27**	O323	eBioscience	100	**CD3**	SP34-2	BD	100	**CD27**	O323	eBioscience	100
Alexa700	**TNF**α	Mab11	BD	50	**FoxP3**	PCH101	eBioscience	50	**TNF**α	Mab11	BD	50

Table S2. Antibody panels for multiparameter flow cytometry
