# Supplementary material for: Critical Loss of the Balance between Th17 and T Regulatory Cell Populations in Pathogenic SIV Infection
Source: PLoS Pathog. 2009 Feb 13;5(2):e1000295. doi: 10.1371/journal.ppat.1000295 (PMC2635016; doi:10.1371/journal.ppat.1000295)
Supplement: Text S1 — This text provides information related to the low frequency and absolute number of CD4+ T cells in the AGMs, as presented in Figures 1E and S1E. (0.05 MB DOC) [file ppat.1000295.s007.doc]

**Text S1:** *This text provides information related to the low frequency and absolute number of CD4*^+^ *T cells in the AGMs, as presented in Figures 1E and S1E.*

The low frequency and absolute number of circulating CD4^+^ T cells in this cohort (of 10-year-old AGMs) reflect normal age-related changes, as documented in a large (n=70) cohort of SIV-infected and -uninfected AGMs of varying ages (from 2 and 15 years) and origins (including Western Africa, the Caribbean, and colonies in the US originating from the Caribbean) (data not shown). For example, a cross-sectional analysis of 44 AGMs of different ages from the same colony in the US showed that 3-year-old Caribbean AGM have CD4 counts in blood averaging 1100 +/-130 cells/µl (n=10, +/-SEM) and that these levels drop to 550 +/-60 cells/µl (n=10, +/-SEM) by 5 years of age and to 265 +/-50 cells/µl by 10 years of age (n=10, +/-SEM) (data not shown). CD4^+^ T cells in AGMs shows a rapid decline at puberty (5 years) then a slower rate of about 30 CD4 cells per µl of blood per year (cross-sectional analysis on SIV^-^ AGMs and longitudinal analysis on SIV^+^ AGMS). Although this rate of CD4 decline is more rapid than that found in macaques and humans, our experience (and that of the literature) indicates that the absence of pathogenic outcome in SIVagm-infected AGMs is not related to CD4 counts: SIVagm infection in neonates [1], juveniles [2,3], and young [4] or older adults (this study and unpublished observations) is non-pathogenic, even though CD4 counts range from over 2000 cells/µl (in neonates) to less than 50 cells/µl (in some 15-year-old animals). Finally, we also documented the frequency of Th17 cells, Treg, and multifunctional cells in 44 AGMs from 3 to 15-years old and the frequency of IL-17 cells and FoxP3^+^ CD4^+^ T cells fall into the normal range for this species and is similar to the levels found in PT macaques at baseline in this study (data not shown).

**REFERENCES**

1. Beer B, Denner J, Brown CR, Norley S, zur Megede J, et al. (1998) Simian immunodeficiency virus of African green monkeys is apathogenic in the newborn natural host. J Acquir Immune Defic Syndr Hum Retrovirol 18: 210-220.

2. Diop OM, Gueye A, Dias-Tavares M, Kornfeld C, Faye A, et al. (2000) High levels of viral replication during primary simian immunodeficiency virus SIVagm infection are rapidly and strongly controlled in African green monkeys. J Virol 74: 7538-7547.

3. Kornfeld C, Ploquin MJ, Pandrea I, Faye A, Onanga R, et al. (2005) Antiinflammatory profiles during primary SIV infection in African green monkeys are associated with protection against AIDS. J Clin Invest 115: 1082-1091.

4. Pandrea I, Apetrei C, Dufour J, Dillon N, Barbercheck J, et al. (2006) Simian immunodeficiency virus SIVagm.sab infection of Caribbean African green monkeys: a new model for the study of SIV pathogenesis in natural hosts. J Virol 80: 4858-4867.
