# Supplementary material for: Critical Loss of the Balance between Th17 and T Regulatory Cell Populations in Pathogenic SIV Infection
Source: PLoS Pathog. 2009 Feb 13;5(2):e1000295. doi: 10.1371/journal.ppat.1000295 (PMC2635016; doi:10.1371/journal.ppat.1000295)
Supplement: Text S2 — This text adds information related to T cell subpopulations as presented in Figures S1F and S1G. (0.04 MB DOC) [file ppat.1000295.s008.doc]

**Text S2**: *This text adds information related to T cell subpopulations as presented in Figures S1F and S1G:* To determine whether and to what extent T cell subpopulations might be altered in composition after SIV infection of the AGM and the PT, CD4^+^ and CD8^+^ T cell populations from peripheral blood and lymph node were divided into subpopulations of naïve (CD45RA^+^CD27^+^), memory (CD45RA^-^CD27^+^), terminally differentiated effector (CD45RA^+^CD27), and effector (CD45RA^-^CD27^-^) T cells (Figure S1G). Such markers have been used by others in macaques [1] and AGMs [2] and have been confirmed by multiparameter flow cytometry using other markers (e.g., CCR7, CD28, CD95, and CD11a). The low frequency of naïve CD4^+^ T cells (CD45RA^+^CD27+) in this cohort is likely the result of age-dependant declines of CD4+ naïve cells. By example, 3-year-old AGMs have >65% naïve CD4+ T cells) (data not shown). When analyzed as a function of time before and after SIV infection in the peripheral blood, PTs showed a relative (but not significant) contraction of circulating memory CD4^+^ T cells (blue) and a relative increase in the fraction of circulating CD4^+^ T effector cells (red, orange) by day 45+, whereas there was a contraction of CD4^+^ T effector cells in the lymph node at this time (Figures S1F and S1G). However, changes in the composition of subset CD4^+^ and CD8^+^ T cell populations over the course of SIV infection didn’t reach statistical significance in PTs and in AGMs by permutation analysis compared to baseline (SPICE software, p>0.05). By contrast, AGMs have over 80% CD4^+^ T cells in the memory pool (blue), and did not show any change in CD4^+^ T cell subpopulations (Figure S1G-upper). In the CD8^+^ T cell compartment, such changes were not observed as a function of time post-infection (Figure S1G-lower).

**REFERENCES**

1. Gauduin MC, Yu Y, Barabasz A, Carville A, Piatak M, et al. (2006) Induction of a virus-specific effector-memory CD4+ T cell response by attenuated SIV infection. J Exp Med 203: 2661-2672.

2. Holznagel E, Norley S, Holzammer S, Coulibaly C, Kurth R (2002) Immunological changes in simian immunodeficiency virus (SIV(agm))-infected African green monkeys (AGM): expanded cytotoxic T lymphocyte, natural killer and B cell subsets in the natural host of SIV(agm). J Gen Virol 83: 631-640.
