# Supplementary material for: Critical Loss of the Balance between Th17 and T Regulatory Cell Populations in Pathogenic SIV Infection
Source: PLoS Pathog. 2009 Feb 13;5(2):e1000295. doi: 10.1371/journal.ppat.1000295 (PMC2635016; doi:10.1371/journal.ppat.1000295)
Supplement: Text S3 — This text provides information related to the characterization of CD3+ CD4−CD8− double negative cells (DN) as presented in Figures S1E and S1F. (0.05 MB DOC) [file ppat.1000295.s009.doc]

**Text S3:** *This text provides information related to the characterization of CD3^+^ CD4^-^CD8^-^ double negative cells (DN) as presented in Figures S1E and S1F.*

A population of CD3^+^CD4^-^CD8^-^ “double negative” (CD3^+^DN) cells was observed in peripheral blood, lymph node, and colon of AGMs prior to SIV infection (Figure S1E), as previously reported [1,2,3]. These cells were TCRγ/δ^-^, C1d^-^ (data not shown), and phenotypically and functionally comparable to CD4^+^ T cells with respect to CD45RA/CD27 subpopulations, cytokine secretion, and the expression of other markers of differentiation and activation (Figure S1F and data not shown), as observed in a large (n=70) cohort of SIV-infected and -uninfected AGMs of varying ages (from 2 and 15 years) and origins (including Western Africa, the Caribbean, and colonies in the US originating from the Caribbean) (data not shown). In contrast to the rapid age-related decline of circulating CD4^+^ T cell in AGMs (Text S1), DN CD3^+^ cell counts were steady with about 300-400 cells/µl in blood from 3 to 15 years old (SIV^-^ AGMs) (data not shown). After SIV infection of the AGM, the fraction and absolute number of this population did not change substantially in any of these sources or in the colon (Figure S1E and data not shown). In the PTs, on the other hand, CD3^+^DN cells were observed only at low frequency (2-5%) in lymph node and PBMCs before infection and modestly increased (to 3-8%) after infection (data not shown and Figure S1E).

**REFERENCES**

1. Murayama Y, Amano A, Mukai R, Shibata H, Matsunaga S, et al. (1997) CD4 and CD8 expressions in African green monkey helper T lymphocytes: implication for resistance to SIV infection. Int Immunol 9: 843-851.

2. Murayama Y, Mukai R, Inoue-Murayama M, Yoshikawa Y (1999) An African green monkey lacking peripheral CD4 lymphocytes that retains helper T cell activity and coexists with SIVagm. Clin Exp Immunol 117: 504-512.

3. Holznagel E, Norley S, Holzammer S, Coulibaly C, Kurth R (2002) Immunological changes in simian immunodeficiency virus (SIV(agm))-infected African green monkeys (AGM): expanded cytotoxic T lymphocyte, natural killer and B cell subsets in the natural host of SIV(agm). J Gen Virol 83: 631-640.
