# Supplementary material for: Critical Loss of the Balance between Th17 and T Regulatory Cell Populations in Pathogenic SIV Infection
Source: PLoS Pathog. 2009 Feb 13;5(2):e1000295. doi: 10.1371/journal.ppat.1000295 (PMC2635016; doi:10.1371/journal.ppat.1000295)
Supplement: Text S4 — This text provides information related to Figure 4, A and B and Figure S2 related to the dynamic of Th1 and Th2/Treg CD4+ T cell populations as well as to SIV antigen-specific CD4+ and CD8+ T cell responses. (0.06 MB DOC) [file ppat.1000295.s010.doc]

**Text S4:** *This text provides information related to* *Figure 4, A and B* *and Figure S2 related to the dynamic of Th1 and Th2/Treg CD4*^+^ *T cell populations as well as to SIV antigen-specific CD4^+^ and CD8^+^ T cell responses.*

The frequency of multifunctional Th1 and Th17 CD4^+^ T cell populations was analyzed in detail. PTs showed a decreased frequency of multifunctional IL-2^+^ Th1 CD4^+^ T cells (expressing TNFα^+^IFNγ^+^ or TNFα^+^ alone) (Figure S2A-upper, R1 and R2, respectively), an increased frequency of monofunctional IFNγ^+^ Th1 (TNFα^-^IL-17-MIP1β^-^) CD4^+^ T cells (Figure S2A-upper, R3) and no change in the frequency of monofunctional IL-2^+^ (TNFα^-^IFNγ^-^IL-17-MIP1β^-^) CD4^+^ T cells (Figure S2A-upper, R4). Conversely, AGMs showed a stable frequency of multifunctional IL-2^+^ Th1 CD4^+^ T cells (expressing TNFα^+^IFNγ^+^) (Figure S2A-lower R1) and an increased frequency of monofunctional IL-2^+^ (TNFα^-^IFNγ^-^IL-17-MIP1β^-^) CD4^+^ T cells (Figure S2A-lower, R4). Overall, acute SIVagm infection in PTs resulted in the reduction of the pool of multifunctional Th1 (IL-2^+^) CD4+ T cells and an increase in the pool of monofunctional IFNγ^+^ Th1, despite the maintenance of IL-2+ monofunctional cells. Conversely, AGMs maintained the pool of multifunctional Th1 (IL-2^+^) CD4+ T cells and increased the frequency of monofunctional IL-2^+^ CD4^+^ T cells. Th17 depletion was also observed at the multifunctional level (IL-2, TNFα) in PT, with all subsets being depleted or negligible in PTs (Figure S2A-upper, A, B and C). Conversely, the pool of multifunctional Th17 cells was maintained (Figure S2A-lower, A and C) or increased (Figure S2A-lower B) in AGMs.

Collectively, the reduction of IL-2-expressing cells in PTs (Figure 4A) was associated with the loss of both Th17 cells (Figure S2A-upper, A, B) and of multifunctional IFNγ-producing (+/-TNFα^+^) Th1 cells (Figure S2A-upper, R1 and R2), at the time when monofunctional IFNγ+ and MIP1β+ (Th1) CD4+ T cells were generated (Figure S2A-upper, R3 and Figure 4, A and D). Changes of this type did not occur in the AGMs.

Although a subset of Th17 cells expressing both IFNγ and IL-17 has been described in patients with Crohn’s disease [1,2], no change in the frequency of IFNγ^+^IL-17^+^ Th17 cells was observed in either species (Figure 3B, Figure S2A and data not shown). Altogether, these results point to the increased destruction and/or decreased production of Th17 cells over Th1 cells and Th2 cells in the SIV-infected PT, not observed after SIV infection of the AGM.

Finally, to determine directly if antigen-specific anti-SIV responses were differently induced in PT and AGM or of a different nature, multiparameter flow cytometry was used to detect and to quantify cells expressing a Th1/Th17 phenotype (e.g., expressing IL-17, MIP1β, IL-2, TNFα, and/or IFNγ) or a Th2/Treg phenotype (e.g., expressing IL-4, IL-10, and/or TGFβ) in various peripheral blood CD4^+^ and CD8^+^ T cells and their memory/effector subpopulations (see Table S2 for staining panels). These responses were elicited by T cell stimulation with autologous SIVagm.sab92018 antigens (e.g., peptide pools of p27(Gag), Gag without p27, Env gp120, Env gp41, and Tat, provided by NIH AIDS Research & Reference Reagent Program or recombinant p27(Gag) produced in the lab), with polyclonal activators as a positive control (e.g., concanavalin A) or not stimulated (RPMI media) as a negative control. Low magnitude (<1%), antigen-specific Th1 CD4^+^(IL-2, TNFα, MIP1β) and CD8^+^ (MIP1β, IFNγ, TNFα, IL-2) T cell responses were detected against each SIV antigen in both species by day 10 and sustained until necropsy (days 45+) (data not shown). In the AGM, there was a trend towards more Th1 multifunctional Env-dominant antigen-specific responses whereas the PT showed more monofunctional Gag-dominant responses (data not shown). When summed for all SIV antigens (Gag and Env), responses oscillated in a 0.1-2.5% range depending upon animal variation and time-points, with no significant differences between PTs and AGMs. Moreover, neither Th17 (IL-17) nor Th2/Treg (IL-4, IL-10, TGFβ) anti-SIV antigen-specific responses were detected during the course of this study (data not shown). Overall, this analysis suggested that the type and the breadth of the anti-SIVagm antigen-specific T cell responses generated against SIVagm during acute infection were dominantly of a Th-1 type. In addition, this antigen specific anti-SIV T cell response was mostly comparable in AGMs and PTs, as found in studies in chronically-infected sooty mangabeys [3,4].

**REFERENCES**

1. Annunziato F, Cosmi L, Santarlasci V, Maggi L, Liotta F, et al. (2007) Phenotypic and functional features of human Th17 cells. J Exp Med 204: 1849-1861.

2. Haider AS, Lowes MA, Suarez-Farinas M, Zaba LC, Cardinale I, et al. (2008) Identification of cellular pathways of "type 1," Th17 T cells, and TNF- and inducible nitric oxide synthase-producing dendritic cells in autoimmune inflammation through pharmacogenomic study of cyclosporine A in psoriasis. J Immunol 180: 1913-1920.

3. Dunham R, Pagliardini P, Gordon S, Sumpter B, Engram J, et al. (2006) The AIDS resistance of naturally SIV-infected sooty mangabeys is independent of cellular immunity to the virus. Blood 108: 209-217.

4. Wang Z, Metcalf B, Ribeiro RM, McClure H, Kaur A (2006) Th-1-type cytotoxic CD8+ T-lymphocyte responses to simian immunodeficiency virus (SIV) are a consistent feature of natural SIV infection in sooty mangabeys. J Virol 80: 2771-2783.
